# Position-dependent effects of RNA-binding proteins in the context of co-transcriptional splicing

**DOI:** 10.1038/s41540-022-00264-3

**Published:** 2023-01-18

**Authors:** Timur Horn, Alison Gosliga, Congxin Li, Mihaela Enculescu, Stefan Legewie

**Affiliations:** 1 Institute of Molecular Biology (IMB), Ackermannweg 4, 55128 Mainz, Germany; 2 University of Stuttgart, Department of Systems Biology and Stuttgart Research Center Systems Biology (SRCSB), Allmandring 31, 70569 Stuttgart, Germany

**Keywords:** Computer modelling, Stochastic modelling, Robustness, Differential equations, Numerical simulations

## Abstract

Alternative splicing is an important step in eukaryotic mRNA pre-processing which increases the complexity of gene expression programs, but is frequently altered in disease. Previous work on the regulation of alternative splicing has demonstrated that splicing is controlled by RNA-binding proteins (RBPs) and by epigenetic DNA/histone modifications which affect splicing by changing the speed of polymerase-mediated pre-mRNA transcription. The interplay of these different layers of splicing regulation is poorly understood. In this paper, we derived mathematical models describing how splicing decisions in a three-exon gene are made by combinatorial spliceosome binding to splice sites during ongoing transcription. We additionally take into account the effect of a regulatory RBP and find that the RBP binding position within the sequence is a key determinant of how RNA polymerase velocity affects splicing. Based on these results, we explain paradoxical observations in the experimental literature and further derive rules explaining why the same RBP can act as inhibitor or activator of cassette exon inclusion depending on its binding position. Finally, we derive a stochastic description of co-transcriptional splicing regulation at the single-cell level and show that splicing outcomes show little noise and follow a binomial distribution despite complex regulation by a multitude of factors. Taken together, our simulations demonstrate the robustness of splicing outcomes and reveal that quantitative insights into kinetic competition of co-transcriptional events are required to fully understand this important mechanism of gene expression diversity.

## Introduction

Splicing is a key step in eukaryotic gene expression that is catalyzed by a large macromolecular complex, the spliceosome. During messenger RNA (mRNA) maturation, the spliceosome removes non-coding parts of the pre-mRNA (introns) and joins together the remaining parts (exons) that form the protein-coding mRNA. Spliceosome assembly is initiated by the binding of U1 and U2 small nuclear ribonucleoproteins (snRNPs) to splice sites. Subsequently, U4-U6 and a large number of protein factors are recruited to yield mature, catalytically active spliceosomes^1–3^. In alternative splicing, different splice products are generated from the same pre-mRNA precursor in a regulated fashion. In the most common mode of alternative splicing, so-called cassette exons are either included or not (skipped) in the final mRNA^4^. Alternative splicing allows for the production of different proteins with different functionalities from the same gene and contributes to proteome complexity^5–7^. Mis-regulated alternative splicing may also lead to the production of non-functional protein isoforms or may cause protein downregulation, e.g., by introducing alternative poly-adenylation sites, shifting the open reading frame, or promoting nonsense-mediated decay^8,9^. As a consequence, changes in alternative splicing may contribute to severe diseases such as cancer or neurodegenerative diseases^10,11^. A deep mechanistic understanding of alternative splicing is therefore needed to develop therapies^10–13^, such as through the identification of new targets for cancer immunotherapy^14,15^, which can provide strategies to combat cancer therapy resistance^16^.

From a systems point of view, splicing is a complex process that requires the exact definition of splice sites on the transcript and their correct joining. Splice site recognition, particularly in alternative splicing, is strongly regulated by RNA binding proteins (RBPs). These bind to cis-regulatory sequence elements in the pre-mRNA, and enhance or suppress spliceosome recruitment to splice sites^7^. For instance, *cis*-acting intronic and exonic splicing silencers (ISS and ESS) are regulatory sequence motifs that bind splicing repressor proteins, e.g. heterogeneous nuclear ribonucleoprotein (hnRNPs) that typically prevent the recruitment of U1 and U2 to nearby splice sites^17,18^. Similarly, intronic and exonic splice enhancers (ISE and ESE) have been discovered, typically as binding sites for the serine-argine repeat (SR) protein class of splicing activators^19–21^. However, the functions of RBPs on splicing are not always so clearly defined, as several RBPS show antagonistic effects, i.e., either promoting or suppressing the inclusion of an exon, depending on their binding location relative to the regulated splice sites^18^. Such functional dependence on the binding position has been evidenced for several splicing regulatory proteins, including hnRNPs^18,22^, SR proteins^21,22^, CELF2^23^, Nova^24^, RbFox^25^, TIA^26^, and PTB^27^, but the underlying molecular mechanisms remain incompletely understood.

There is strong evidence that splicing mainly occurs co-transcriptionally in human cells, and that transcription and splicing mutually influence each other by spatial and kinetic coupling mechanisms^28–31^. Spatial coupling arises because both processes share molecular components and therefore occur in close proximity. For instance, RNA polymerase II (Pol II) contains the C-terminal heptad repeat domain (CTD) of the large subunit that is required for the deposition of splicing factors to splice sites^32–34^. In addition, kinetic coupling occurs since the speed of pre-mRNA transcription determines how fast downstream splice sites become available to compete with alternative upstream splice sites^35^. Furthermore, the rate of transcript elongation affects the formation of secondary structures in the pre-mRNA, and thereby the accessibility of splice sites for splicing factors^30^.

For cassette exons, a strong dependence of the inclusion frequency on transcription velocity has been reported^31^. For instance, slow Pol II elongation may increase the time window for the recognition of weak exons, leading to their higher inclusion^25^. However, in contrast, slow Pol II elongation can also favor exon skipping by promoting the recruitment of inhibitory RBPs that prevent exon recognition^29^. In genome-wide experiments, four different classes of exons have been identified based on their Pol II velocity dependence, including monotonically increasing or decreasing exon inclusion with Pol II speed (see above), but also bell- and U-shaped behaviors, with the latter two classes accounting for approximately 50% of velocity-sensitive genes^31^. In the latter scenarios, fast and slow Pol II mutants shift the splicing outcome in the same direction, suggesting that for these exons, the spliceosome operates at an optimal point for physiological Pol II values.

Various strategies have been employed to quantitatively model the impact of *cis*-regulatory sequence features on alternative splicing outcomes. These approaches range from automated machine learning based on transcriptome-wide splicing data or from synthetic libraries^36–39^ to mechanistic descriptions of splicing reaction kinetics^35^^,^^40–48^. Mechanistic modeling studies are typically focused on certain splicing decisions and have naturally favored minimizing complexity, given the limited amount of available experimental data. Therefore, they often described splicing as a quasi-post-transcriptional process, i.e., all relevant splice sites are assumed to be available when splicing decisions are reached^42,43,46,47^. While this assumption could be consistent with co-transcriptional splicing on the elongating transcript, it fails to explain why alternative splicing outcomes are affected by the transcript elongation rate. Therefore, other mechanistic models explicitly consider that upstream splicing sites are present earlier than others, implying that the corresponding splicing decisions are kinetically favored, in particular at slow elongation rates^35,44,45,48^. For instance, one recent study has sought to explain how co-transcriptional splicing has impacted gene structure and evolution, focusing on genomic level predictions^45^. Another work employed a kinetic model of co-transcriptional splicing to accurately predict the combined impact of both the position and quantity of ESEs or ESSs on the splicing of engineered designer exons^41^.

Here, we build upon this previous modeling work on co-transcriptional splicing regulation and mechanistically describe how spliceosomes assemble on the elongating transcript, and how a trans-acting RBP modulates the process in a time- and position-dependent manner by binding to cis-regulatory sequence elements. Crucially, we extend the description of co-transcriptional kinetics to include the availability and binding of *cis*-regulatory motifs, in addition to splice sites. We show that simple kinetic models account for several non-intuitive behaviors including the existence of optimal RNA polymerase speeds for the inclusion of alternative exons. We additionally demonstrate mechanisms by which a single protein can both increase and decrease inclusion of an exon. These findings suggest that substantial interplay exists between the various regulatory mechanisms of alternative splicing. This will be important for informing a complete understanding of splicing, and the development of interventions in splicing decisions.

## Results

### Modeling of co-transcriptional alternative splicing regulation

**Fig. 1:**
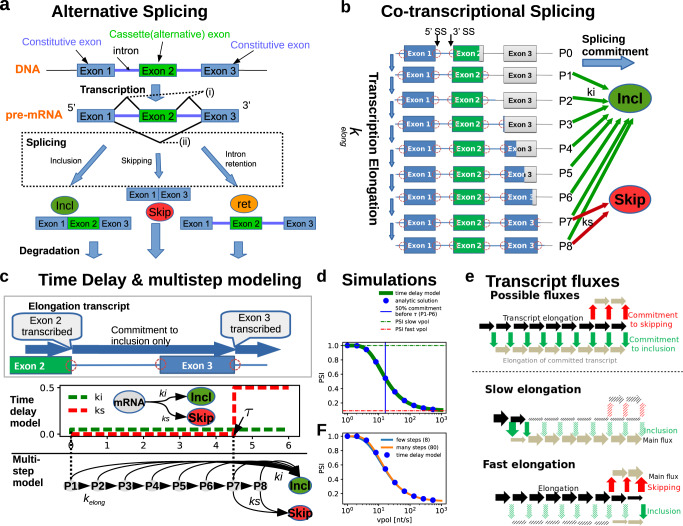
Modeling co-transcriptional splicing of a three-exon gene. **a** Schematic representation of alternative splicing. After transcription, the nascent pre-mRNA is spliced to remove introns (thin lines) and eventually exons (boxes, E1-E3). The middle alternative exon can either be included (i) or skipped (ii) (i.e., spliced out), or the introns are retained if splicing is unsuccessful. **b** Illustration of co-transcriptional splicing during successive elongation of the nascent transcript (indicated by the expanding coloration of introns and exons in vertical direction). Considering an exon definition mechanism (see main text), introns are spliced out only after completion of synthesis of both flanking exons. Thus, splicing of the first intron (and thus commitment to inclusion; green arrows) is possible once exon 2 is fully synthesized, whereas commitment to skipping (red arrows) requires Ex3 synthesis. **c** Delay and multi-step modeling of co-transcriptional splicing. Both models describe transcript elongation only after exon 2 synthesis is complete, as no splicing is possible earlier (panel **b**). The delay model consists of three species (unspliced mRNA as well as skipping and inclusion isoforms) and the skipping rate (ks) increases in a step-wise manner at a time delay τ (red dashed line), whereas inclusion (ki) takes place immediately (green line). τ represents the time it takes to complete exon 3 synthesis, and thus reflects the elongation speed. In the multistep model, the transcript elongation is implemented using a chain of elongation states (P1-P8) and a progression rate kelong. In P1-P6, only commitment to inclusion is possible (ki), whereas both splicing reactions (ki, ks) are possible in P7-P8 (see also **b**). Intron retention is neglected in both models. **d** and **f** Relationship between polymerase elongation speed (vpol) and splicing outcome, as measured by the PSI metric (percent spliced-in, PSI = incl/(incl+skip)). In **d**, numerical simulations of the delay model (solid green line) are compared to an analytical solution of the same model (blue dots; Methods), whereas in **f** numerical solutions of delay and multistep models (with few or many elongation steps) are shown (see legend). The value of vpol was calculated based on the length of exons and introns in the transcript, and based on τ (delay model) or the parameter kelong (multistep model), see Methods. Horizontal lines in **d** show the inclusion-to-skipping ratio (ki/ks) before and after the delay τ. The vertical blue line marks *vpol* at which a share of *50%* of the pre-mRNA is spliced before τ. **e** Schematic representation of splicing fluxes in the model (top), alongside with dominant fluxes for extremely slow and fast RNA polymerase speed *vpol* (middle and bottom, respectively). Thick solid arrows show the main reaction fluxes, whereas shaded arrows typify minor or non-existent fluxes.

To model the dynamics of splicing, we investigated the behavior of a minimal system, in which an alternatively spliced cassette exon is flanked by introns and outer constitutive exons (Fig. 1a). Alternative splicing in this system involves the inclusion or exclusion of the middle cassette exon. Additionally, if splicing fails, one or both of the introns may be retained and intron retention isoforms are generated.

The scheme in Fig. 1a represents a scenario of post-transcriptional splicing regulation, as splicing decisions are made only after pre-mRNA synthesis is complete. Co-transcriptional splicing regulation involves an additional level of complexity, since not all splice isoforms can be generated at the same time (Fig. 1b). Early after transcription initiation, no splicing commitment is possible, as the necessary sequence elements still need to be synthesized (State P0). Specifically, in human cells, introns can only be spliced out after both flanking exons are fully recognized by the spliceosome, a mechanism that has been termed exon definition^46,49–51^. Thus, the completion of exon 2 synthesis (state P1) marks the first time point in the lifetime of a transcript at which intron 1 splicing is possible. In contrast, the competing skipping reaction occurs later in the transcript lifetime, once all introns and exons are fully synthesized (States P7-P8). Therefore, commitment to the exon 2 inclusion isoform is possible at earlier stages (States P2-P8) than the commitment to skipping (States P7-P8).

To numerically simulate co-transcriptional splicing, we implemented a system of ordinary differential equations (ODEs). The time shift of skipping relative to inclusion was implemented by a time delay τ for the skipping reaction (Fig. 1c, “time delay model”; Supplementary Figure 1). Specifically, the rate of commitment to skipping (ks) is initially zero and increases in state P7 to a positive value. In contrast, the rate of commitment to inclusion (ki) is time-invariant if we neglect the initial splicing-less phase after transcript initiation and start the simulations after the completion of exon 2 synthesis (State P1 in Fig. 1b). For simplicity, we neglected possible intron retention scenarios. Thus, intron 1 splicing was assumed to be always accompanied by intron 2 splicing and therefore marks the commitment to inclusion.

This system using time-dependent reaction rates was solved by integrating the ODEs in a stepwise manner: initially, all transcripts are assumed to simultaneously initiate elongation, i.e., the mRNA precursor was set to 1, whereas the inclusion and skipping products are zero. Then, the integration was performed by considering only the commitment reaction to inclusion until the time point τ where skipping starts. Afterwards, both reactions were taken into account and the concentrations of skipping and inclusion after long integration times reflect the probabilities for commitment to the corresponding splicing products. Under the assumption of steady-state gene expression and equal degradation rates for skipping and inclusion products, this probability is proportional to the experimentally measurable concentrations of the splicing isoforms (see Methods for details).

### Slow elongation favors exon inclusion in the basic model

We analyzed how the incidence of skipping and inclusion isoforms changes with varying transcript elongation rates (Fig. 1d). Specifically, we asked whether variations in RNA polymerase speed (vpol) affect the inclusion frequency of the cassette exon in the model. Such a dependency of splicing outcomes on transcript elongation velocity had been reported in the published experimental literature^28,29,31,52,53^.

To mimic altered transcript elongation rates, we assumed that the delay parameter τ for the skipping reaction resulting from transcript elongation is inversely proportional to vpol (see Methods). As a measure of the splicing outcome, we monitored the PSI metric (PSI = inclusion/(inclusion + skipping)), which ranges between 0 and 1 for no and full inclusion, respectively. In line with an earlier modeling study and experimental work on co-transcriptional splicing, we find that the inclusion frequency decreases with increasing polymerase elongation rate (Fig. 1d)^25,28,45^. At low polymerase speed, inclusion is the only splicing outcome, since all transcripts commit to inclusion before the transcript is elongated beyond the third exon, where skipping can occur (Fig. 1c, d and e). In contrast, fast transcript elongation eliminates this kinetic advantage of inclusion, and the splicing outcome is determined by the relative commitment rates of skipping and inclusion, as in the post-transcriptional scenario (Fig. 1a; see Methods). As the value of vpol increases towards infinity, the model converges towards post-transcriptional results as the delay between splicing commitment events decreases towards 0. Using analytical calculations, it can be shown that the PSI-elongation curve always decreases monotonically (Methods; Supplementary Figure 2), and as such is incapable of recapitulating more complex PSI profiles such as observed in Fong et al.^31^ In line with kinetic competition between inclusion and elongation, the drop in PSI (measured as the inflection point) occurs when the skipping delay due to polymerase progression (τ) is comparable to the time scale of the inclusion reaction (τ ≈ 1/ki, Fig. 1d).

To quantitatively confirm the above simulation results, we considered an alternative implementation of co-transcriptional splicing regulation: in this multistep model variant, we described the progression of RNA polymerase using multiple consecutive elongation states (each represented by one ODE) and assumed that commitment to skipping is possible only late in the elongation chain (Fig. 1c, bottom). Alterations in the transcript elongation speed were simulated by changing the progression parameter between states (k_elong_). When plotting PSI as a function of the resulting polymerase speed (vpol; see Methods), we found that the simulation results of the multistep model quantitatively agreed with the delay model for a sufficiently large number of elongation steps (Fig. 1f). This confirms the expectation that a multistep chain with many elongation steps approximates a hard delay well, whereas a chain with few steps only yields a qualitative agreement (Fig. 1f). Taken together, two distinct methods exist for modeling co-transcriptional splicing which both yield identical results provided that enough reaction steps are considered in the multistep formulation.

### Non-canonical splicing responses to elongation encoded by position of RBP binding

**Fig. 2:**
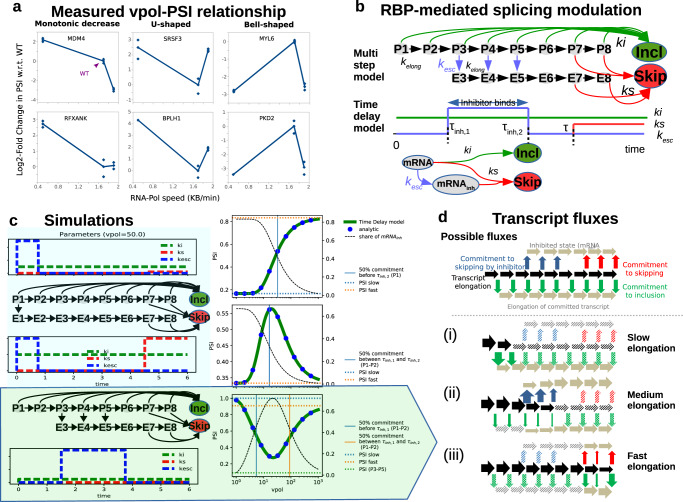
Complex PSI-vpol profiles with RBP-mediated splicing inhibition. **a** Experimentally measured relationship between polymerase speed (vpol) and splicing outcomes (PSI) taken from Figs. 2, 3, and 4, and supplemental Figs. 3, 4, and 5 of Fong et al.^31^. Splicing profiles of selected splicing events (indicated in titles) as assessed by PCR and gel electrophoresis are shown for polymerase mutants with enhanced and lowered transcript elongation rate (left and right datapoints), alongside profiles when cells express the wildtype enzyme (middle datapoints). Each datapoint is one out of three replicate measurements. The PSI metric can increase or decrease with polymerase speed, and may show U- or bell-shaped responses. **b** Co-transcriptional splicing commitment model with RBP-mediated inhibition of exon inclusion. Top: Multistep model with inhibitor-mediated escape from inclusion (*k*_esc_) towards skipping-committed pre-mRNA molecules (species E3-E8). The depicted model topology involves two steps before inhibition/escape reaction (P1-P2), a window of opportunity for inhibition (P3-P5), a late state (P6) where skipping is not possible (exon 3 not synthesized) and stages where both inclusion and skipping reactions are possible (P7-P8). Bottom: The time delay model with RBP-mediated inhibition derived from the topology in Fig. 1c. Compared to the basic model it contains an additional species *mRNA*_*inh*_ representing an RBP-inhibited *mRNA*. Also there are two additional time delays τ_inh,1_, τ_inh,2_ marking the window of opportunity for inhibition. **c** Simulated PSI-vpol profiles with RBP-mediated inhibition. Top and middle: Early inhibition scenario, where RBP-mediated inhibition (*k*_esc_) occurs already before/when exon 2 is synthesized (reaction P1 → E1 in multistep model). Monotonically increasing or bell-shaped PSI-vpol profiles are observed, depending inclusion-to-skipping (ki/ks) ratio towards the end of the transcript (red dashed lines in PSI-vpol diagram). Bottom: Late inhibition scenario, in which the RBP-mediated inhibition (kesc) occurs after synthesis of exon 2 (reactions P3 → E3, P4 → E4 and P5 → E5 in multistep model), can result in a U-shaped PSI-vpol profile. The black solid lines depict the fraction of *mRNA* being inhibited during transcription (i.e., progressing through the mRNA_inh_ state in the delay model). This fraction decreases since fast elongation diminishes the window-of-opportunity for RBP binding. Horizontal lines indicate approximate splicing outcomes predicted by the ratio ki_i_/(k_s_ + k_esc_) in different splicing commitment regimes along the transcript. As discussed in panel **d**, at slow and fast elongation, splicing decisions are made early and late after transcript initiation, respectively. Therefore, PSI slow, fast and P3 → P5 indicate inclusion-to-skipping ratios at early, late and intermediate times in transcript lifetime. Vertical lines depict the value of vpol at which 50% splicing commitment occurs in these regimes along the transcript. **d** Schematic representation of transcript fluxes at different elongation rates for the late inhibition scenario (bottom panel in **c**). On the top all possible fluxes for one mRNA molecule are depicted. The lower part shows the main reaction flux considering three elongation regimes. The solid arrows show the main reaction flux, the shaded arrows typify minor or not existing fluxes.

Genome-wide measurements revealed that changes in the transcript elongation speed affect splicing in a gene-specific manner^29,31^. Fong et al. analyzed global splicing patterns in cells expressing fast and slow RNA polymerase mutants. In line with the simulations above, they found that a large number of genes show the canonical response where slow elongation shifts splicing towards inclusion (Figs. 1d and 2a, left). However, exons also frequently show the inverse behavior, where a slow RNA pol speed promotes skipping^29,31^. Moreover, two additional gene classes exist, in which the relationship between transcript elongation and PSI is non-monotonous, resulting in the bell- or U-shaped curves in the experimental splicing-elongation (PSI-vpol) diagram (Fig. 2a, middle and right). These complex behaviors are impossible to obtain with simple kinetic competition of inclusion and skipping as depicted in Fig. 1d and f, requiring additional factors to be considered.

Dujardin et al. proposed a mechanistic explanation for the inverse splicing response, where slow elongation promotes exon skipping^29^: They experimentally showed that this response is caused by an RBP that inhibits exon inclusion through competition with a downstream U2AF2 binding site, where RBP binding is favored by slow RNA Polymerase delaying the synthesis of the competing U2AF2 binding site. To better characterize this mechanism, we extended our model of co-transcriptional splicing regulation, and additionally considered an RBP-mediated inhibitory reaction (k_esc_) which shifts the mRNA into an inhibited state (mRNA_inh_; Fig. 2b, bottom). In this state, commitment to inclusion is no longer possible, but skipping can still occur, though only after the delay time τ. In similarity to the basic model, τ reflects the time it takes for polymerase to complete the synthesis of the last exon. In essence, the RBP inhibitor introduces early commitment to skipping, while preventing inclusion.

Interestingly, this extended model of co-transcriptional splicing regulation not only explained monotonic PSI-vpol diagrams, but could also realize bell- and U-shaped curves depending on the chosen kinetic parameter values (Fig. 2c). As experimental literature has previously demonstrated that many proteins preferentially or exclusively bind co-transcriptionally^54–56^, we assumed that the inhibitory RBP must be deposited in a limited time window by the elongating polymerase. This assumption is also mathematically consistent with the model of Dujardin et al., if we use the simplifying assumption that the binding rate of the competing reaction (U2AF2 binding in Dujardin et al.^29^) is much greater than binding of the inhibitory RBP. In our model, we implemented this by restricting the inhibitory reaction k_esc_ to a time window between the delay times τ_inh,1_ and τ_inh,2_ (Fig. 2b; Supplementary Table 1). This time frame (τ_inh,1_ → τ_inh,2_) reflects the time it takes for elongating RNA polymerase to reach and pass the position of the RBP motif within the pre-mRNA sequence. Therefore, the parameters τ_inh,1_ and τ_inh,2_ are proportional to the assumed polymerase speed and the time window τ_inh,1_ → τ_inh,2_ increases for slow elongation.

Whether the PSI-vpol diagram is monotonically increasing, decreasing, U- or bell-shaped critically depends on the initial delay of inhibitor binding (τ_inh,1_): In the regime of very slow elongation delayed reactions play no role, since splicing decisions are made just after (the very long) elongation cycle has started. For such slow elongation, strong and instantaneous inhibitor binding (τ_inh,1_ = 0) favors skipping, whereas inclusion is the only outcome if inhibitor binding is delayed (τ_inh,1_ > 0). Thus, monotonically increasing or bell-shaped PSI-vpol diagram can be observed for τ_inh,1_ = 0 (Fig. 2c, top and middle rows), while decreasing or U-shaped curves occur otherwise (Fig. 2c, bottom; Supplementary Table 2). In terms of pre-mRNA sequence, the no delay scenario (τ_inh,1_ = 0) locates the RBP binding motif to (or upstream of) the alternative exon, whereas a delay would correspond to RBP binding downstream of the alternative exon. Hence, in the model, the position of the inhibitory RBP binding motif in the transcript has strong qualitative effects on splicing outcomes.

Non-monotonous (U- or bell-shaped) behavior in the PSI-vpol-diagram requires three clearly distinguishable splicing regimes at different elongation rates, as schematically depicted in Fig. 2d for the U-shaped case: slow elongation favors a splicing decision early in the transcript elongation cycle and inclusion is the only possible outcome (Fig. 2d, i). At medium elongation rates, the transcript on average elongates further until a splicing decision is made, and in this regime inhibitor-mediated skipping is the dominant splicing outcome (Fig. 2d, ii). Finally, at very fast elongation transcription is finished before splicing commitment, and skipping dominates over inclusion for the chosen kinetic parameters in this quasi-post-transcriptional regime. (Fig. 2d, iii). As a result, the PSI-vpol diagram exhibits a U-shape, as shown in Fig. 2c alongside with the corresponding splicing commitment rates (left, bottom). Similar arguments of kinetic competition between (i) generating a splicing decision at a certain length of the elongating transcript vs. (ii) elongating further explain other shapes the PSI-vpol diagram (Fig. 2c, top and middle).

Taken together, co-transcriptional splicing outcomes are shaped by the relative rates of skipping and inclusion which dynamically change during the transcript elongation due to changes in: (i) the availability of exons for splicing; (ii) inhibitor-mediated commitment to certain splicing fates. By an appropriate choice of these parameters, gene-specific PSI-vpol diagrams may be realized as reported experimentally^29,31^.

### Mechanistic modeling of RBP-mediated modulation of splicing

So far, we made simplifying assumptions about the effects of the inhibitory RBP on splicing outcomes. To confirm our findings in a more realistic setting, we turned to mechanistic modeling of RBP binding to pre-mRNA and effects on splicing decisions.

**Fig. 3:**
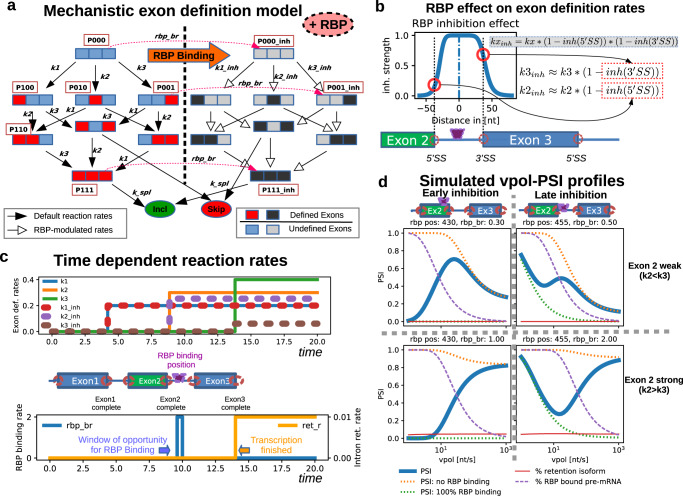
Mechanistic modeling of spliceosome binding and exon modulation by RBPs. **a** Exon definition model of co-transcriptional splicing. In the unbound pre-mRNA (P000) each exon can be cooperatively bound by the spliceosome and this is modeled using lumped and irreversible exon definition reactions (rate constants k_1_-k_3_). Definition of the 1^st^, 2^nd^ and 3^rd^ exon switch P000 to the states P100, P010, P001, respectively. Spliceosome binding affects splicing outcomes (modeled by the splicing reaction k_spl_), as the states P101 (exon 2 undefined) and P111 (exon 2 defined) give rise to skipping and inclusion, respectively, whereas no splicing (but intron retention) is possible otherwise. The inhibitory RBP irreversibly binds to unspliced pre-mRNA states (transition from left to right subnetwork; e.g., P000 → P000_inh), affects spliceosome binding (reduced rates k1_inh-k3_inh), but splicing rates remain unchanged (k_spl). See also panels **b** and **c**, and Supplemental Material for details. **b** Local effects of RBP binding on exon definition rates (k_1_-k_3_). Not all three exon definition rates (k_1_-k_3_) are reduced by RBP binding, but only those of exons located nearby the RBP binding site (indicated by magenta pictogram and vertical dashed line at x = 0). Inhibition is modeled by a bell-shaped inhibition function (kx_inh_), in which the RBP effects on k_1_-k_3_ decay within + /−50 bp around the binding site. **c** Implementation of co-transcriptional splicing using time-dependent reaction rates. Shown are the reaction rates as a function of time after transcript initiation, with step-like increases or decreases corresponding to time delays. The schematic representation of the three-exon minigene indicates the correspondence of time delays and position of elongating RNA polymerase within the gene. Each exon can be defined immediately after its transcription is complete (top), RBP binding occurs in a short time window after elongation across the RBP binding site and retention is possible only after transcription is finished. **d** Simulated PSI-vpol profiles for RBP binding within exon 2 (left) or in the downstream flanking intron (right) using the local inhibition profile depicted in B. Two simulations were performed for each RBP localization, in which either exon 2 is less well recognized than exon 3 (k2 < k3, top), or vice versa (k2 > k3, bottom). The PSI profiles (thick solid lines) can be considered as weighted sum of two limiting cases, a model without RBP-mediated inhibition (green dashed lines) and one in which the RBP binds with high efficiency to ~100% of the transcripts (orange dashed lines). At low and high polymerase velocities, the PSI is approximated by strong and no RBP binding, respectively, as slow elongation extends the temporal window of opportunity for polymerase-mediated RBP-recruitment. The fraction of RBP-bound to the pre-mRNA in the full model (solid line) is shown as a dashed magenta line. The abundance of the retention isoform is shown as solid red line.

This mechanistic model was based on our previous work on exon definition, in which we modeled recruitment of pioneering spliceosome U1 and U2 subunits to splice sites (Fig. 3a and Enculescu et al.^46^). Specifically, we considered that all three exons may be “defined” by cooperative U1 and U2 binding. Initially, the pre-mRNA is synthesized as an unbound precursor (P000), and then irreversible exon definition may occur by rate constants k1-k3 (Fig. 3a, left). For instance, both outer exons are defined in the states P101 and P111, whereas the middle exon is either undefined (P101) or defined (P111). These spliceosome binding patterns impact splicing outcomes, as we assume splicing reactions (k_spl_) lead to inclusion (state P111), skipping (P101) or retention (all other states). The resulting mathematical model is a limit case of the more general kinetic model introduced in Enculescu et al.^46^ if we assume irreversible spliceosome binding to the pre-mRNA. However, in contrast to the previous work^46^, we additionally considered here dynamic changes in splicing outcomes due to RBP inhibitor binding and co-transcriptional splicing dynamics.

As depicted in Fig. 3a (right), the inhibitory RBP modulates splicing outcomes by irreversibly binding to all unspliced pre-mRNA states (e.g., P000 → P000_inh) with the rate *rbp_br*. Subsequently, spliceosome binding occurs at reduced rates (k1_inh-k3_inh), but splicing rates (k_spl_) remain unchanged. Thus, the inhibitory RBP changes splicing outcomes by blocking initial spliceosome recruitment. Importantly, this occurs only locally around the sites of RBP binding, i.e., around an assumed RBP motif. The spatial range of RBP effects on spliceosomes which we implemented in the model is depicted in Fig. 3b. We assumed a bell-shaped inhibition profile, in which the RBP effects on k1-k3 decay within ~100 bp around the binding site, as previous experimental literature has shown a substantial decay in a protein’s effect when a protein’s binding site is shifted from 70 to 140 or 200 bp from the splice site^57,58^.

Co-transcriptional spliceosome binding and splicing were considered in the model by assuming that an exon can only be defined after its synthesis is complete. Hence, the rate constants k1-k3 and k1_inh-k3_inh increase in a stepwise manner after transcript initiation with individual delays reflecting the relative positions of exons within the pre-mRNA (Fig. 3c; Supplementary Table 3). For RBP binding, we again assumed RNA polymerase-dependent recruitment^55^, and therefore modeled RBP binding to be restricted to a short window-of-opportunity, reflecting the phase when the elongating enzyme pass the RBP motif (Fig. 3c, bottom). Since most human transcripts are spliced co-transcriptionally^52^, we assumed that full-length transcripts may undergo a transition into an intron retention isoform with the rate ret_r (Fig. 3c, bottom).

For the kinetic parameters, we assumed physiologically plausible ranges in our simulations: Values for the RNA polymerase velocity (vpol) were chosen based on the quantitative data in the experimental literature^59^. Taking the polymerase speed into account, the delay times for exon definition reactions (i.e., waiting times for spliceosome binding to splice sites) were adjusted based on the intron/exon structure of a previously published reporter gene comprising RON exon 10-12^46^ The subsequent spliceosome binding and splicing commitment reactions were chosen to reflect the experimentally reported overall splicing times ranging from a few seconds to several minutes^48,59,60^. In further support for the physiological plausibility of our model, we demonstrate in Supplementary Figure 3 (also see Supplementary Table 4) that it is able to quantatively reproduce dynamic co-transcriptional splicing measurements at the single-molecule level reported by Coulon et al.^59^.

The mechanistic model fully reproduced the experimentally observed PSI-vpol diagrams, including monotonically decreasing or increasing curves, as well as bell- and U-shapes (Fig. 3d). In summary, a monotonic decrease is observed in the absence of an inhibitory RBP (Fig. 3d, dotted orange lines in all panels). If the RBP binds early during the elongation cycle (i.e., within the alternative exon) this behavior can be reversed into a monotonic increase or a bell-shape (Fig. 3d, left), whereas binding downstream of the alternative exon allows for the U-shape (Fig. 3d, right). In the model, all these behaviors are linked to dynamic changes of RBP inhibitor binding at different elongation rates (Fig. 3d, dashed pink lines in all panels). Another important determinant is the relative strength of exons 2 and 3 (i.e., the ratio of the respective recognition parameters k2 and k3): A reduced exon 2 recognition rate favors skipping, especially for fast elongation (post-transcriptional case), and may therefore convert a monotonically increasing curve (Fig. 3d, bottom left) into a bell-shape (Fig. 3d, top left).

Taken together, our mechanistic model describes co-transcriptional splicing regulation at the level of individual splice site regulation by RPBs. Compared to the simple model (Figs. 1 and 2), the mechanistic description can accommodate more complex PSI-vpol diagrams (e.g., Fig. 3d, top right) and shows more diverse behavior for a given binding position of the RBP (Fig. 5). Furthermore, it better represents the biophysical properties of RBP and spliceosome binding, and therefore allows us to better characterize mechanisms of splicing regulation by RBPs.

### Complex position-dependent RBP effects in the mechanistic model

In the experimental splicing literature, extensive evidence supports that the same RBP can frequently act as both activator and inhibitor of exon inclusion depending on its location of binding (see Introduction): For instance, experiments in which a variety of RNA binding protein motifs were placed up- or downstream of a 5ʹ splice site showed opposite effects on splice site usage depending on their position^22^. This effect was dependent on which protein was being investigated; with SR and traditional activator proteins having an enhancing function on alternative exon recognition upstream, and a silencing function downstream of the 5ʹ splice site, whilst the opposite was observed for hnRNPs and traditional silencer proteins.

**Fig. 4:**
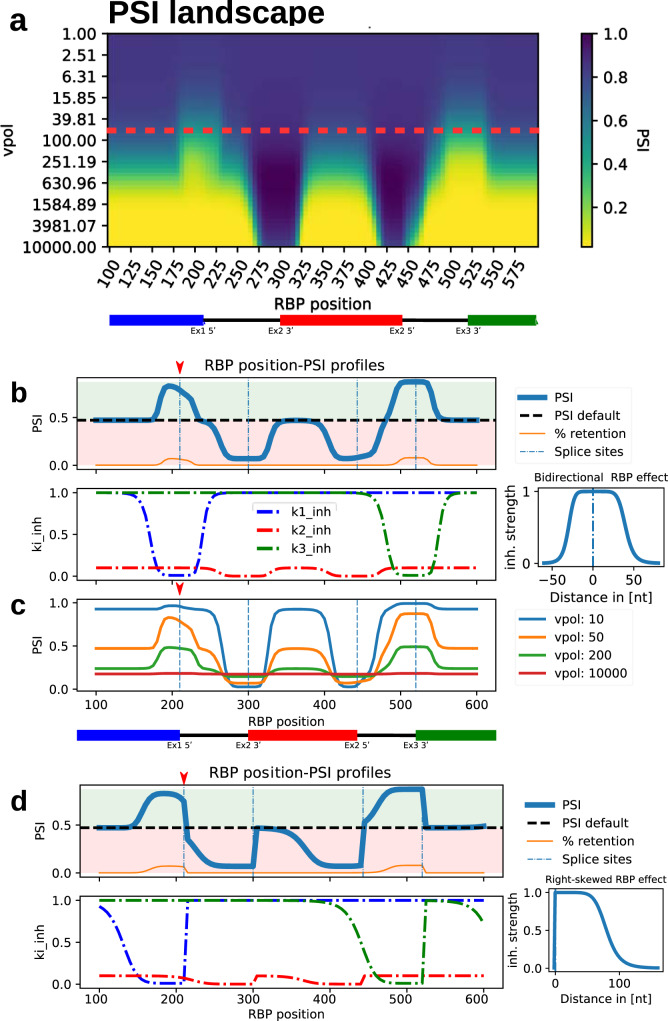
Splicing activation and inhibition by RBPs depending on their binding position. **a** Heatmap of simulated PSI values as a function of the position of the RBP binding motif (x-axis) and RNA polymerase speed vpol (yaxis) using the inhibition function shown in Fig. 3b. The schematic representation below indicates the positions of the three exons (colored rectangles) and the joining introns (black lines). The horizontal red dashed line indicates the vpol value chosen in **b**. **b** Position-dependent opposing RBP effects on alternative splicing. Top: PSI (blue line) and intron retention (orange) as a function of the RBP binding position at a fixed RNA polymerase speed (*vpol* = *50 nt/s)*. Depending on its binding position (and the inhibited exons), the inhibitory RBP can promote or suppress the inclusion of the cassette exon relative to a simulation without RBP (horizontal black dashed line, ‘PSI default’). Increased inclusion is accompanied by slightly increased retention proportion due to a delay in exon 1 or 3 recognition and splicing. Bottom: Modulation of protein bound exon definition parameters (k1_inh-k3_inh) by the RBP binding position. The local RBP inhibition function is assumed to be bidirectional around the RBP binding position (x = 0) as depicted on the right (same function as the one shown in Fig. 3b). **c** Dual RBP role most pronounced at intermediate RNA polymerase velocities. Simulations with the same parameters and assumptions as in **b**, but for varying RNA polymerase speeds (see legend). Strong activation and inhibition of exon inclusion depending on the RBP position are most pronounced at intermediate polymerase velocities (orange line). Outer plateau at RBP position ~150 corresponds to the absence of RBP-mediated inhibition. **d** Position-dependent RBP effects on exon inclusion for asymmetric RBP-mediated inhibition of splice sites. Top: An asymmetric, right-skewed RBP inhibition function (right) switches the directionality of splicing changes (activation vs. inhibition) when the RBP binding is located upstream or downstream of a 5ʹ splice site of an exon (vertical dashed lines). Bottom: The splicing switch from exon inclusion to skipping (or vice versa) is accompanied by a sharp intersection of constitutive and alternative exon definition rates (red arrows).

Using our mechanistic co-transcriptional splicing regulation model, we investigated how the RBP binding position affects splicing outcomes (PSI) for a given polymerase elongation speed (vpol). We generated PSI heatmaps, in which we systematically varied vpol and the position of the RBP binding motif, again using the percent spliced-in metric as a readout (Fig. 4a; Supplementary Table 5). For the relative length of introns and exons, we chose the dimensions of a minigene spanning RON exons 10-12 which we characterized in our recent work^43^. In line with the published literature, we found that an inhibitory RBP which blocks spliceosome recruitment can be both an inhibitor and an activator of alternative exon inclusion depending on its binding position. This can be seen along the red dashed line in Fig. 4a and in the corresponding two-dimensional projection in Fig. 4b (top): here, inhibitor binding close to splice sites of constitutive exons increases inclusion (Fig. 4b, vertical dashed lines around positions 210 and 530, respectively). In contrast, inclusion is diminished for inhibitor binding around the splice sites of the alternative exon (Fig. 4b, vertical dashed lines around positions 300 and 440, respectively). These inclusion levels should be compared to the plateaus of peripheral RBP binding (positions around 100 and 575), which correspond to a lack of RBP impact on exon definition rates *k1_inh* – *k3_inh* (Fig. 4b, bottom). Thus, in our model the RBP can play a dual role, being both an activator and inhibitor of inclusion depending on its binding position.

From a mechanistic viewpoint, this dual role can be explained by kinetic competition at the level of exon 2 and exon 3 definition: In the context of co-transcriptional splicing regulation, suppressing the outer exons (lowering k1 or k3) by local RBP binding gives the middle alternative exon a longer time window to be recognized by the spliceosome. Thus, RBP binding favors recognition of all three exons and thus inclusion when compared to skipping (which requires only definition of exons 1 and 3). In contrast, local inhibition of the alternative exon (k2) selectively blocks the inclusion reaction, thereby lowering the PSI relative to the absence of RBP-mediated regulation. These position-dependent RBP effects resemble experimental observations for PTB, an RBP that indeed inhibits inclusion when bound to the alternative exon, while promoting inclusion when located to flanking constitutive exons^27^. Interestingly, in our model, the position-dependence disappears for very fast elongation, since the RBP is less likely to be deposited by a rapidly progressing polymerase enzyme (Fig. 4c, red line). This further demonstrates the complex interplay of RBP binding position and transcript elongation in the context of co-transcriptional splicing.

Another free parameter in the model is the spatial RBP-mediated inhibition profile which we modelled using a bidirectional + /−50 bp Hill function around the RBP binding motif in Fig. 4b (right). Figure 4d shows simulations with an alternative, right-skewed inhibition function, where the RBP mainly affects downstream sequence elements that are yet to be transcribed. For this inhibition function, the RBP position-dependent effects on PSI better match the experimental reports for RBPs other than PTP, in which RBP binding within the alternative exon (positions 300-440) had the opposite effect compared to downstream binding (positions >440) (see Introduction).

Interestingly, both the bidirectional and the right-skewed RBP inhibition scenarios exhibit a local asymmetry in their impact on PSI, in particular at the 5ʹ splice site of the 1^st^ exon (red arrows in Fig. 4b, c and d). Here, the RBP has a maximal impact on PSI when bound upstream of the effected 5ʹ splice site, whereas downstream binding diminishes the RBP effect. This asymmetric RBP effect arises from kinetic competition and temporal order of events during transcript elongation: upstream RBP binding can saturate the transcript before exon 1 definition is possible, thereby effectively preventing the definition reaction. In contrast, for a downstream RBP binding site exon 1 definition may be partially complete before recruitment of the inhibitory RBP.

**Fig. 5:**
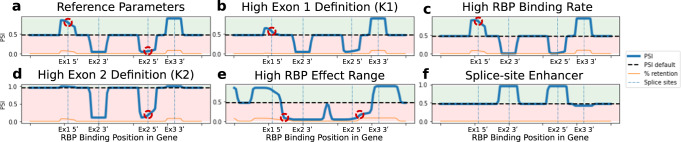
Kinetic competition of exon definition and RBP-mediated inhibition around splice sites. **a** Reference simulation for the position-dependent RBP effect. Figure 4b has been recreated with an increased Hill coefficient of the RBP’s range-dependency function (Fig. 4b, right) from 8 to 32 (see Methods, Eq. 15**)**. This results in the RBP exhibiting a quasi-binary inhibition profile as a function of the binding site distance. Due to kinetic competition of exon definition and RBP-mediated inhibition, the RBP effect on PSI is asymmetric up- and downstream of the 5′ splice sites of exons 1 and 2 (see red circles and main text). **b** and **d** Multiplication of the exon definition rate parameters k1 and k2 by 5 relative to the reference simulation lowers the local magnitude of the RBP effect, while increasing RBP effect asymmetry around the relevant 5′ splice sites (see red circles), since exon definition more efficiently competes with RBP-mediated inhibition. **c** Multiplication of the RBP binding rate (rbp_r) by 4 slightly increases the RBP effect on PSI, with slightly more pronounced saturation when binding occurs upstream of 5′ the splice site of exon 1 (red circle). **e** Increasing the RBP effect range from 25 nucleotides (nt) to 65 nt shows that the asymmetric regulation of PSI by the RBP around 5′ splice sites persists if the RBP has overlaying effects on multiple splice sites (red circles). **f** The RBP’s function (Eq. 14) was altered by adding, rather than subtracting, the inhFunc term, therefore switching it to an activating function. When the RBP functions as an enhancer (activator) of splice site usage, the kinetic competition effect (RBP effect asymmetry on PSI) is not observed around the splice site that the RBP effects. This can be explained by the fact that an activator behaves non-competitively with the definition of the splice sites it impacts, instead enhancing the exon definition rates k1-k3.

To further investigate this kinetic competition, we analyzed PSI profiles for various parameter values against a single set of parameters for comparison (Fig. 5a). In line with kinetic competition, we found that increasing the exon 1 definition rate (Fig. 5b) and the RBP binding rate (Fig. 5c) had opposite effects on the PSI profile around the splice site. In particular, a high exon 1 definition parameter almost completely abolished the impact of the RBP downstream of the splice site, thereby enhancing asymmetry of the RBP effect (red circle in Fig. 5b). Similarly, an increased exon 2 definition parameter resulted in a more asymmetric PSI profile around the 5′ splice site of exon 2 (red circle in Fig. 5d). Hence, the temporal order and relative speed of exon definition vs. RBP-mediated inhibition can be shown to effect the position-dependence of RBP effects in a co-transcriptional context. In our model, asymmetric RBP effects are not observed around the 3′ splice sites of the exons, since we assume that exon definition can only occur once the whole exon has been synthesized. However if the RBP controls splice sites with a sufficiently spatial large range to simultaneously affect a 5′ and 3′ splice site of an intron additional inflection points can be observed (red circles in Fig. 5e). Furthermore, kinetic competition does not apply to splice site activators, as activator binding promotes, rather than competing with, exon definition. Corresponding simulations for an activator confirmed that the impact of an activator on PSI is symmetric, i.e., independent of the binding position relative to the splice site (Fig. 5f).

Taken together, by mechanistic modeling we derived a kinetic framework that quantitatively predicts splicing outcomes in co-transcriptional context based on RBP binding position, elongation rate and exon definition rates.

### Noise in alternative splicing follows a binomial distribution

Cellular RNAs are frequently expressed at low levels, often summing up to a total concentration of only a few molecules per cell^61^. At such low concentrations, biochemical reactions do not occur deterministically, but involve a probabilistic component. Thus, alternative splicing may be a stochastic process with uncertainty in the exon inclusion frequency, as opposed to a deterministic system where the fraction of the inclusion isoform is predictable and completely determined by the kinetic rate constants^62^.

**Fig. 6:**
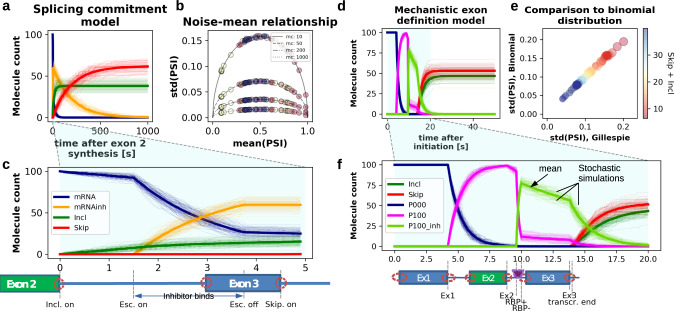
Intrinsic noise in stochastic splicing simulations quantitatively agrees with a binomial model. **a** and **c** Stochastic time course simulations of the splicing commitment model with time delay depicted in Fig. 2c (bottom) using the Gillespie algorithm. The RNA polymerase speed (*vpol*) is set to 50 nt/s and the initial mRNA molecule count to 100. The thin lines show 100 individual simulation runs (each representing one single cell) and the thick lines represent their mean value for each species (see legend). Panel **c** shows only the first few time points of the simulation, with the time delays of individual reactions being annotated at the bottom (Incl.on, Esc.on and Skip.on: inclusion, RBP-mediated escape and skipping reactions switched on, respectively). **b** Noise-mean relationship in the splicing commitment solely depends on average PSI and total molecule count. The mean and std of the PSI-value (*Incl/(Incl* + *Skip)*) at the end of simulation (t = 1000) were calculated for different absolute mRNA numbers (molecule counts, mc). To ensure stability of results, each circle represents the average of 5000 individual simulation runs. Various mean PSI-values were generated by the variation of *vpol* (see Methods for details). The results are compared to a binomial model (thin lines), in which the molecule count is given by the number of trials (see legend). **d** and **f** Stochastic time course simulations of the mechanistic exon definition model depicted in Fig. 3a using the Gillespie algorithm. Parameters correspond to the bottom left panel in Fig. 3d with a polymerase speed of 50 nt/sec and an initial P000 count of 100 molecules. Details are analogous to panels **a** and **c**, but distinct molecular species are displayed, i.e., the inclusion and skipping isoforms as well as the spliceosome binding intermediates P000 and P100 without RBP binding, or with the RBP being present (P100_inh). The time delays of the individual reactions are annotated at the bottom (RBP+ and RBP-: RBP binding switched on and off, respectively). **e** Splicing noise in Gillespie simulations of the exon definition model quantitatively agrees with the binomial model. The PSI noise (standard deviation, std) at the end of a simulation was averaged over 5000 simulation runs, each circle representing a different mean(PSI) value and total molecule count, generated using various parameter values and/or RBP binding positions (see Methods). To correct for varying degrees of retention, the molecule count in the mechanistic model equals the sum of *Incl* and *Skip* isoforms (see colorbar). After matching of mean(PSI) and molecule count, mechanistic and binomial simulations perfectly overlap.

To quantify uncertainties in splicing outcomes, we performed stochastic simulations using our co-transcriptional splicing models (Figs. 1–3). For stochastic simulations, we sampled the time-dependent probability of the exon definition, RBP binding, and splicing reactions from exponential distributions to determine the order of reaction steps using the Gillespie algorithm^63^ (see Methods). This way, we account for stochastic variation in the binding reactions of the RBP and splicing factors, and how this impacts splicing decision making. The stochastic simulation was repeated 5000 times for each parameter combination, each model realization reflecting the behavior of one single cell. Exemplary time course simulations for the splicing commitment model (Fig. 2c) are shown in Fig. 6a and c. Likewise, Fig. 6d and f contain simulations of the mechanistic model analyzed in Fig. 3b and c. At all time points, both models show a simple unimodal distribution of the model species across single cells.

For the splicing commitment model with time delay (Fig. 1c), the cell-to-cell variability of splicing outcomes was quantified by relating the standard deviation and mean of the PSI metric across cells (Fig. 6a and b; Supplementary Table 6). In this analysis, we considered simulations with different initial transcript counts per cell. Furthermore, we took into account various model parameter values (polymerase speeds) as well as model variants with RBP binding at different locations (Fig. 2c). The resulting noise-mean relationship exhibits a bell-shape, showing zero noise at a mean PSI close to one or zero, and a peak at intermediate mean PSI values (Fig. 6b). Interestingly, these curves we observe in response to stochastic variations are very similar to bell-shaped PSI changes induced by mutations or RBP knockdowns^42,43,64^. Thus, the PSI metric exhibits a nonlinear response to both deterministic and stochastic perturbations. This is due to the fact that skipping and inclusion reactions are balanced at intermediate PSI values, whereas one of the reactions strongly dominates at low and high PSIs, respectively.

In the stochastic model, the height of the std(PSI)-peak is solely determined by the total transcript count per cell, but not by the other parameters in the model. At very low molecule numbers, the splicing outcome is very noisy, whereas it approaches the deterministic solution (i.e., shows a small standard deviation) for a total expression of >200 molecules per cell (Fig. 6b). Interestingly, the noise-mean curves of all model variants are perfectly congruent with a simple binomial distribution, in which two categorical outcomes are drawn from a random distribution (solid lines in Fig. 6b). Thus, after correction for the total number of splicing events, the system behaves like a simple binary decision between two alternative isoforms despite being regulated by multiple mechanisms including the elongation rate and RBP binding. The presence of the intron retention isoform in the mechanistic model prevented a similar analysis for this model, so the mean and standard deviation of PSI were compared directly to the binomial model to determine the noise relationship (Fig. 6e). Again, after a consideration of total number of inclusion and skipping molecules per cell, the model perfectly agrees with the predictions of a binomial distribution, even though the splicing decisions are complex events involving multiple exon definition reactions. Taken together, our results show that while co-transcriptional alternative splicing regulation by trans-acting factors increases the number of pathways by which a splicing decision can be made, with substantial effect on outcomes, it adds little intrinsic stochastic noise. This explains why a large part of cell-to-cell variability in two splicing decisions that were experimentally characterized using single-molecule RNA-FISH could be explained by a purely binomial model^65^.

### Bimodality in alternative splicing arises from promoter bursting and feedback

We primarily observed binomial splicing fluctuations in the previous section, however bimodality in alternative splicing has been reported in the literature. Such bimodal behavior is characterized by two clearly separated peaks in the PSI histogram, i.e., either inclusion or skipping predominates, and this may be physiologically relevant, as alternative splicing isoforms have been found to be significant in determining cell identity^66–68^. We therefore studied how bimodal distributions can be realised in our models.

**Fig. 7:**
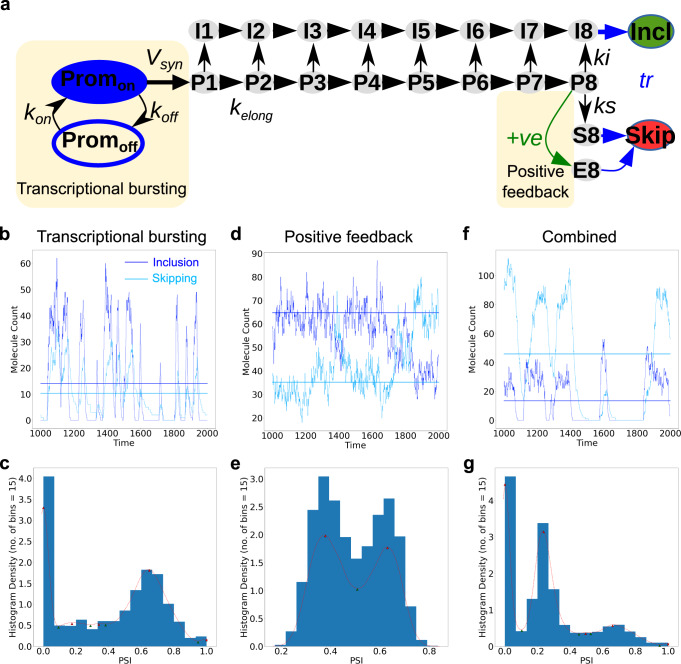
Bimodality in splicing fates due to transcriptional bursting and positive feedback. **a** Extended co-transcriptional splicing model that incorporates transcriptional bursting or/and positive feedback. The promoter randomly switches between a transcriptionally active state (Prom_on_) and an inactive state (Prom_off_), with rate constants k_on_ (inactive to active) and k_off_ (active to inactive). Transcription initiation starts only when the promoter is active (with rate Vsyn). Upon initiation, transcripts begin to be synthesized via RNA polymerase elongation. As in our previous models (Fig. 1c), the elongation is modeled as a multi-step process (P1 → P8) with an elongation rate k_elong_ for each step. Splicing commitment to the inclusion isoform (with rate ki) can occur during elongation (I1 → I8), while commitment to skipping (with rate ks) is only possible later when the last exon is full synthesized. The protein product of the skipping isoform (functioning as an RBP) can bind to its own mRNA precursor to enhance the commitment to skipping isoform (P8 → E8). This positive feedback is mathematically modeled by the +ve function which is detailed in Methods. Emergence of multimodality in splicing arising from transcriptional bursting with variable degradation (**b** and **c**), positive feedback (**d** and **e**), and the combination of both (**f** and **g**). **a**, **c** and **e** represent both stochastic and ODE results at steady state (imepoints 1000 to 2000), starting from an active promoter with zero pre-existing transcripts. Histograms of PSI values in **b**, **d** and **f** represent density of timepoints within each bin over an extended time period of 10,000 timepoints. The red dotted line gives the results of a kernel density estimator based on the normal distribution provided with the Python SciPy Stats module (gaussian_kde, bw_method = “scott“), with local minima and maxima represented by green and red arrows, respectively.

In Fig. 7a we demonstrate the realisation of two possible mechanisms of achieving bimodality in splicing (see also Supplementary Table 7). The first is achieved through transcriptional bursting, in which the promoter of a gene switches between periods of minimal and high transcription^69^. The time course in Fig. 7b shows how the inclusion and skiiping isoforms at increase proportionally to each other during a burst, giving the higher PSI peak in the histogram of the time course (Fig. 7c). We additionally assume different degradation rates for the two splicing isoforms. Then, upon termination of the burst, the unstable isoform (inclusion) decays rapidly, with the slow degrading isoform (skipping) eventually becoming the sole isoform, corresponding to the lower peak at PSI = 0 in the histogram (Fig. 7c). Hence, the differential temporal stability of inclusion and skipping isoforms after burst termination establishes bimodality.

Our second mechanism involves a positive feedback loop, in which the skipping isoform promotes further skipping reactions once the skipping isoform reaches a threshold level. Such positive autoregulation has been shown for the SXL gene in *D. Melanogaster*^70^. As can be seen in Fig. 7d, positive feedback regulation gives rise to alternating periods of high and low PSI, corresponding to separated peaks in the time course histogram (Fig. 7e). Bimodality emerges, because the feedback loop is either essentially off at low levels of skipping, but stochastic fluctuations may switch on the loop, giving rise to plateaus, where skipping exceeds inclusion. Notably, when the feedback is off the system averages to the ODE result (Fig. 7d).

Combining both transcriptional bursting with differential degradation, and positive feedback, results in tri-modality, as observable in Fig. 7f. In this model, the third intermediate peak in the histogram arises because during a sufficiently long burst, skipping accumulation triggers positive feedback, thereby eventually lowering the PSI during the burst. Notably, if the positive feedback loop becomes effective during a burst, it is possible for the effect to persist and impact the starting PSI of a closely following burst, as observed in the two bursts between timepoints 1200 and 1400 (Fig. 7g).

Taken together, these results show how stochastic implementations of our splicing models can be modified to realize bimodal distributions. The underlying mechanisms, transcriptional bursting and feedback amplification of splicing outcomes, are common in human gene expression regulation. Notably, feedback amplification may not only be established by direct positive feedback, but could also involve double negative feedback regulation, which has been described for several splice-regulatory RBPs^71,72^, or in related gene-regulatory networks (e.g., the LIN28-let-7 system^73^). By realizing discrete splice isoform expression regimes of key regulatory molecules, the proposed mechanisms may aid in the establishment of cell identity.

## Discussion

In this work, we derived a quantitative description of co-transcriptional splicing dynamics. We implemented two models that differed in their level of detail: (i) a splicing commitment model, in which effective commitment reactions to skipping and inclusion are assumed. (ii) a detailed mechanistic model, in which skipping and inclusion isoforms are not produced independently, but are interrelated, as both splicing decisions share a common set of constitutive splice sites that need to be recognized.

Both models describe co-transcriptional splicing commitment by assuming that certain reaction steps occur with a delay relative to other events, and thereby resemble a previously proposed mathematical model of co-transcriptional splicing^45^. In addition to these delay models, we also implemented a multistep formulation of co-transcriptional splicing commitment dynamics, in which transcripts of different length are described as discrete states, each state being a variable in the ODE system. By numerical simulations, we show that multistep and delay formulations yield identical results if a sufficient number of steps are considered in the multistep formulation, i.e., if the multistep formulation approaches a continuum and the discretization approximation can be neglected.

While our co-transcriptional splicing commitment models resemble published work^45^, we focus here on a novel aspect, the determination of co-transcriptional splicing outcomes by RBPs. In the splicing commitment model, we made the ad-hoc assumption that the RBP binding blocks commitment to inclusion, and thereby establishes early skipping commitment. In the exon definition model, we considered additional mechanistic details and described co-transcriptional recruitment of the RBP inhibitor to defined pre-mRNA sequences by RNA polymerase and considered local effects on exon definition. Thereby, the RBP simultaneously affects inclusion, skipping and/or intron retention isoforms.

In both co-transcriptional splicing commitment models, the RBP inhibitor could establish non-intuitive splicing responses towards alterations in the RNA polymerase velocity (Figs. 2 and 3). In line with the experimental literature, these responses included monotonically increasing, monotonically decreasing, bell-shaped or U-shaped PSI-vpol relationships^31^. All such behaviors could be recapitulated by the appropriate choices of RBP binding position and splice site strength, with the RBP binding position determining the PSI value at low polymerase speeds, and the splice site strength determining the PSI at high polymerase speeds. Finally, a necessary assumption in the model was that RBP binding (i.e., the percentage of occupied pre-mRNAs) dynamically changes for alterations of the RNA polymerase elongation speed (Fig. 3d). Such a speed dependency may arise if RNA polymerase deposits the RBP on the sequence during elongation when it passes the RBP sequence motif^55^. In this scenario, faster elongation shortens the time window of opportunity for RNP deposition on the pre-mRNA, and thereby affects total RBP binding. In line with the assumptions, RNA polymerase is known to recruit numerous splicing regulatory factors to mRNA, including Prp19^54^, Prp40^56^, and U2AF2^54^. For several of these RBPs, it was shown that binding independent of RNA polymerase is inefficient, suggesting that they are recruited primarily during transcription. Moreover, evidence exists that in this mode of co-transcriptional binding slower transcript elongation enhances overall RBP binding, as we had assumed in our model^29^.

Many RBPs exhibit antagonistic effects depending on their binding position relative to other splicing-related sequence features^18,21–27^. For some proteins such as Rbfox these contradictory effects result from looping and other long-range interactions that are not considered in this work^25^. For several other proteins, however, previous work has shown contradictory effects arising from short-range interactions either around a single splice-site^22,23^ or when there is competition between alternative 5′ or 3′ splice sites^22^. In line with these experimental findings, we observe a dual role of inhibitory RBPs on splicing outcomes in our model, involving suppression or enhancement of inclusion even at a fixed RNA polymerase velocity (Fig. 4). In the present implementation, the underlying mechanism is the kinetic competition of outer and inner exons: in the context of co-transcriptional splicing regulation, inhibitor binding to the outer exons simultaneously reduces skipping and inclusion, but provides a kinetic advantage to the full recognition of all three exons (P111, inclusion) compared to the pure recognition of the outer exons (P101, skipping)^44^. In the literature, alternative mechanisms for position-dependent RBP effects have been suggested, including the formation of distinct spliceosome complexes for upstream and downstream RBP binding^74^. Our model provides a quantitative framework to implement such mechanisms and to design experiments to test them.

Naturally, the action of an RBP on a splice site is effected by the distance between the RBP binding site and the splice site. For simple regulatory mechanisms, reliant on direct interactions instead of topological alterations of the transcript, the magnitude of the effect on splicing decays with splice site distance^57^. We additionally observe changes in the magnitude of the PSI effect of an RBP at the 5ʹ splice sites of exons 1 and 2, with maximal impact for RBP binding upstream of the splice site (Fig. 5). This RBP effect asymmetry arises from kinetic competition of exon definition and RBP-mediated inhibition of the definition reaction, as an increasing number of transcripts will have already undergone exon definition reactions before the RBP binds as the binding site is moved downstream. The RBP effect asymmetry is absent for activators, as these promote and do not compete with the exon definition reaction.

Our models provide a means to design experiments to describe complex relationships between polymerase speed and the percentage inclusion of alternative exons that have previously been observed on a genomic basis^31^, but have thus far been difficult to characterize mechanistically at the level of individual exons. Our models predict that these non-intuitive behaviors arise depending on the position of binding of an inhibitory RBP within or downstream of the alternative exons. As a means to experimentally test these predictions, we propose to introduce artificial RBP binding site into three-exon minigenes, e.g., through shifting of binding motifs^41^, introduction of artificial binding sites using fusion proteins^75^, and tethered-oligonucleotide binding sites^76^. Crucially, our model predicts that placement of an inhibitory RBP upstream of an alternative exon’s splice sites results in a monotonically increasing exon inclusion for increasing polymerase speed, or an optimal polymerase speed for inclusion (Fig. 2). Splicing analysis of the proposed minigenes, e.g., by capillary gel electrophoresis or RNA sequencing, upon systematic perturbation of the transcript elongation rate using polymerase mutants or the topoisomerase inhibitor campothecin^59^, will confirm whether the behaviors predicted by our models indeed occur in a real biological system. In contrast, placement of an inhibitor protein downstream of the alternative exon is predicted to result in a U-shaped relationship with a specific polymerase speed that results in minimal exon inclusion (Fig. 2). Again, this prediction may be tested by combining artificial RBP binding sites with a titration of the RNA polymerase-dependent elongation rate. Taken together, our models represent a framework for designing in vivo testing schemes in order to quantitatively understand effects transcript elongation and RBP binding positions on splicing outcomes. The validation experiments will, in turn, constrain the parameter values and molecular mechanisms considered in the model, thereby resulting in a refined description of co-transcriptional splicing dynamics.

Mathematical models are abstractions of complex biological systems. Likewise, our models of co-transcriptional splicing do not capture the full complexity of the process. Given the limited quantitative experimental data available in the literature, a full description of all biological aspects was also not what we aimed for, since the consideration of additional mechanisms leads to additional unknown parameters and thus to uncertainties in the behavior of the model.

Our goal in the present study was a conceptual understanding of co-transcriptional dynamics and its modulation by RBPs. It is very likely that the main findings in this work will remain qualitatively valid if additional regulatory mechanisms (such as the ones discussed below) are taken into account, although this remains to be determined in future studies, e.g., if sufficient quantitative information becomes for certain aspects of splicing regulation. For the present work, we focused on the most simple model versions that are much easier to handle in terms of simulation analysis due to lower degrees of freedom.

One important simplification we made was the assumption of splicing commitment reactions that do not necessarily reflect the actual splicing catalysis. In fact, experimental work suggests that commitment likely involves the formation of the earliest spliceosomal cross-intron complexes^77^. Importantly, while subsequent spliceosome maturation by recruitment of U4-U6, followed by two-step catalysis (intron removal) and finally spliceosome release could be implemented in the model, this would not affect splicing outcomes, as long as the initial commitment step is (quasi-)irreversible and rate-limiting. Another important assumption in our model is 100% strict exon definition (i.e., both splice sites of an exon are either jointly defined or not), as this considerably reduces the number of spliceosome binding states to 8 (Fig. 3a), as opposed to 64 binding states that would arise if each exon would be characterized by two independently defined splice sites^46^ (‘intron definition’). According to the biological literature, the U1 subunit is first recruited to the 5′ splice and then cooperatively stimulates the subsequent recruitment of U2 to the 3′ splice site^43^. In the model, we assumed very strong cooperativity to reduce the number of model parameters, given that we recently showed that this assumption allows for a quantitative description of splicing outcomes in a large-scale mutagenesis dataset for the RON minigene^46^. However, it should be noted that in our model we could reflect a continuum of mechanisms, ranging from pure exon definition (perfect cross-exon cooperativity) to pure intron definition (no cross-exon cooperativity).

Splicing frequently occurs recursively, implying that many introns are removed progressively in multiple reaction steps^78,79^, and not in a single step as we assumed in our model. Again, the consideration of recursive splicing would result in a substantial increase in model complexity, as each intron removal step would have to be combined with all other possible elongation, commitment and protein binding states in the transcript, possibly exhibiting its own specific splicing parameter. The present model is well suited to describe systems with recursive splicing if the consecutive removal of intron parts is characterized by a single rate-limiting step. If there is no single rate-limiting step, the models of co- vs. post-transcriptional splicing need to be modified to take into account that the kinetics of intron removal do not exhibit simple exponentially distributed waiting times, but rather peaked waiting times that are a hallmark of multistep processes.

Alternative splicing in regulated in various ways including RNA structure, epigenetic regulation, differential expression of RBPs, sequence mutations, cellular ATP content and many others^80–83^. In our modeling approach, we did not represent each of these mechanisms, but focused on the fundamental control points of splicing regulation on which these mechanisms converge, i.e., RNA polymerase speed and RBP-mediated regulation. Importantly, our simulations of altered polymerase speed in fact reflect various biological mechanisms including RNA structure, epigenetic regulation and altered ATP content. Likewise, changes in the total RBP concentration in the model may reflect altered RBP expression or reduced RBP binding due sequence mutations and/or altered structure in the pre-mRNA. Our model is ready to be extended to describe any of the upstream regulatory mechanisms in detail if the required quantitative experimental data becomes available. For instance, in our recent work we showed that it is possible to infer the effect of thousands of point mutations in the RON minigene on exon definition and splicing outcomes using a model similar to the one presented in this work^46^. Using such mutational data, RNA structure prediction algorithms^84^ may be applied to establish links between RNA secondary structure and splicing outcomes. In fact, a quantitative model such as the one presented here may help to infer how structural elements in the RNA impact on RBP binding affinity and splice site recognition strength. Epigenetic chromatin marks such as DNA methylation play an important role in splicing regulation and exon recognition, mainly by affecting the RNA pol velocity and thereby transcript elongation^85,86^. Based on systematic perturbations of an epigenetic modification, e.g., by epigenome editing, accompanied by global splicing analyses (RNAseq), it might be possible to quantitatively model the impact on vpol and splicing outcomes in future studies. Hence, our conceptual model of co-transcriptional splicing regulation serves as a starting for the detailed analysis of specific subsystems of co-transcriptional splicing regulation, besides providing general insights into the principles of the process.

Finally, we converted our co-transcriptional splicing models into a stochastic formulation to investigate cell-to-cell variability in splicing arising from intrinsic stochastic fluctuations. Surprisingly, our mechanistic splicing model (Fig. 6) shows noise behavior that is fully consistent with a minimal binomial sampling, even though we considered complex splicing mechanisms including co-transcriptional dynamics, multistep commitment to splicing and RBP-mediated regulation. In fact, some experimentally characterized splicing decisions could be well approximated by the binomial model^65,87^, whereas others showed higher noise levels and/or were even characterized by a bimodal distribution^67,68^, in which individual cells show high or low but never intermediate inclusion levels. In Fig. 7, we explore the ability of extended model variants to realize bimodal PSI distributions. Bimodality can be achieved through transcriptional bursting with differing isoform lifetimes, which might occur if one of the isoforms is subjected to nonsense-mediated decay, or exhibits alternative 3ʹ untranslated regions and polyadenylation. Bimodality can also be realized through the implementation of a positive-feedback loop, such as occurs in the SXL gene in *D. Melanogaster*^70^. Positive-feedback loops behave equivalently to double-negative feedback loops involved in cell fate decisions, such as those observed in the LIN28-let-7 system^73^, the nSR100-REST system^72^, and SFSR2-MBD2 system^71^. In our implementation of feedback, only a single isoform is necessary for bimodality in its absolute expression level, which is in keeping with widespread reporting of coupling between alternative splicing and nonsense-mediated decay as a means of controlling expression levels in auto-regulated splicing events^88^. Taken together, these experimental and theoretical results suggest that the binomial case is the default splicing outcome, but that specific splice-regulatory mechanisms allow for deviations from it. In the future, it will be interesting to further extend our models to see which additional mechanisms increase stochastic fluctuations in splicing outcomes. For instance, a deviation from binomial behavior may be observed if: (i) reversibility of spliceosome binding to splice sites is considered, or (ii) noise arising from long-term RBP expression fluctuations is taken into account^89^.

In conclusion, our mechanistic splicing models are valuable toolboxes to test competing hypotheses for alternative splicing regulation at the cell population and single-cell levels. They cover a large number of experimental perturbations including sequence mutations, RBP knockdowns/knockouts, artificial recruitment of RBPs, modulation of splicing by antisense oligonucleotides and alterations of polymerase elongation rates. The mechanistic model described in Fig. 3 comprises four kinetic parameters (k1-k3, k_spl_) in the absence of RBP-mediated regulation and four additional kinetic parameters in the presence of an RBP (k1_inh-k3_inh, rbp_br). Other free parameters (delays and RBP binding positions) are mainly set by the gene structure, so that it seems feasible to calibrate gene-specific mechanistic models by fitting to genome-wide datasets (RNAseq, SLAMSeq) under multiple perturbation conditions (see also Davis-Turak et al.^45^). Such global analyses may provide mechanistic insights into the coordinated regulation of multiple splice isoforms and thereby into the general principles of splicing regulation in health and disease.

## Methods

### Splicing commitment model - time delay implementation

In Figs. 1, 2 and 5a-c, we performed simulations of the splicing commitment model using time delay model, in which we describe the splicing fate of a transcript during its synthesis and consider splicing reactions that eventually occur with delays.

We implemented a system of four ordinary differential equations (ODEs) describing unspliced transcripts (*mRNA*), spliced transcript with the alternative exon included (*Incl*) or skipped (*Skip*) and inhibited mRNA (*mRNAinh*), in which the unspliced mRNA is bound by an inhibitory RBP and inclusion is no longer possible (Fig. 1b).

1 $$\begin{array}{ll}\frac{d}{{dt}}mRNA & = - mRNA \cdot \left( {kesc + ki + ks} \right) \\ \frac{d}{{dt}}Incl &= ki \cdot mRNA\\ \frac{d}{{dt}}Skip &= ks \cdot \left( {mRNA + mRNAinh} \right) \\ \frac{d}{{dt}}mRNAinh & = kesc \cdot mRNA - ks \cdot mRNAinh \end{array}$$

All splicing commitment reactions are assumed to irreversible and occur with the parameters ki (inclusion), ks (skipping) and kesc (“escape” reaction: inhibitor-mediated commitment to skipping). To implement co-transcriptional splicing, we consider that splicing commitment reactions can occur after different delay times (see below), and chose these delay times based on known molecular mechanisms of splicing. Specifically, we assumed an exon definition mechanism which is known to apply for most splicing events in human cells^49,50,90^. In exon definition, not only the spliced intron, but also the flanking exons need to be fully recognized by the spliceosome for intron splicing to occur. Hence, during transcript elongation, splicing of the first and second intron is only possible after synthesis of exon 2 and 3 is complete, respectively.

For simplicity, we neglect the initial, splicing-less phase before exon 2 is fully synthesized (State P0 in Fig. 1b), and model only the transcript fate afterwards (State P1 in Fig. 1b). In terms of splicing commitment, exon inclusion is immediately possible after start of the simulation, whereas skipping can only occur only later, once both introns and all exons have been synthesized (States P7-P8 in Fig. 1b). To implement the time shift of skipping relative to inclusion, we did not explicitly model polymerase progression, but considered a time delay τ for the skipping reaction (Fig. 1b, “time delay model”). Specifically, the rate of commitment to skipping (k_S_) is initially zero and then increases in a step-like manner, whereas the commitment rate to inclusion (k_i_) is time-invariant.

To numerically implement these time delays, we performed our simulations in several consecutive simulation steps, each of which represents the interval between two time delays. The simulation starts at time *t* = *0* (completion of exon 2 synthesis, see above) and we set all species to zero, except for the unspliced *mRNA*, which we assume to be 1. Thus, we assume a synchronized population of *mRNA* molecules (100% just elongated through exon 2), and will use the following numerical simulation routine to calculate the relative probability to end up in a certain splicing fate.

The ODE system is initially integrated until the first time delay using ODE solver *odeint* from the phyton package *scipy (v. 1.3.1)*, subpackage *integrate*^91^. In Fig. 1c and D, no skipping can occur in this first time phase (ks = 0), and inhibitor-mediated skipping does not occur (kesc = 0). Thus, the ODE system reduces to:

2 $$\begin{array}{*{20}{c}} {\frac{d}{{dt}}mRNA = - ki \cdot mRNA} \\ {\frac{d}{{dt}}Incl = ki \cdot mRNA} \end{array}$$

The concentrations of the simulated *mRNA* and *Incl* species at *t* = *τ* represent the likelihood of a transcript to be unspliced or spliced to inclusion until the time delay *τ*, i.e., until exon 3 is fully synthesized. To determine the final fate of all transcripts, we continue the simulation for *t* > *τ*, now considering that skipping is possible.

3 $$\begin{array}{ll} \frac{d}{{dt}}mRNA &= mRNA \cdot \left( {ks + ki} \right) \\ \frac{d}{{dt}}Skip &= ks \cdot mRNA \\ \frac{d}{{dt}}Incl &= ki \cdot mRNA \end{array}$$

The initial value vector of species in this second time phase is the final species vector from the first integration step (Eq. 3) at the end time point *t* = *τ*. The simulation is performed till the unspliced mRNA species approaches zero, i.e., until all molecules are spliced. At the end time point of merged simulation $t \to \infty$ (in practice t = 10000), the value of *mRNA* is very close to zero, and the values *Incl*_*t→∞*_ and *Skip*_*t→∞*_ represent the probability for the corresponding isoforms to be produced from the precursor.

Alternative splicing is quantified using the PSI metric, which equals the probability of inclusion.

4 $$PSI = \frac{{Incl_{t \to \infty }}}{{Incl_{t \to \infty } + Skip_{t \to \infty }}} = Incl_{t \to \infty }$$

In Fig. 2, the RBP inhibitor prevents inclusion and this is implemented using the “escape” reaction *kesc* which results in early transcript commitment to skipping. It is assumed that inhibitor binding and early skipping commitment can occur in a restricted time window between the delays *τ*_*inh,1*_ and *τ*_*inh,2*_. Thus, when considering this inhibitor-mediated skipping, the number of consecutive integration intervals increases to four (Fig. 2b). For each time phase, there are different effective sets of constants and ODEs, as summarized in Supplementary Table 1.

The following explanations are based on Supplementary Figure 1.

The time delays described above represent the time it takes for RNA polymerase to elongate through the gene body until distinct splicing decisions are possible. The delays are therefore effective elongation parameters, that depend on the dimension of introns, exons and the RBP binding motif (“window-of-opportunity”) relative to the total length of the transcript as well as the speed of RNA polymerase which may be specific for each gene^92^. Specifically, each time delay inversely proportional to the RNA polymerase velocity (*vpol*), given in nucleotides per second,

5 $$\begin{array}{ll} \tau _{inh,1} &= \frac{{tr_{len}}}{{l \cdot vpol}} \cdot k, \\ \tau _{inh,2} &= \frac{{tr_{len}}}{{l \cdot vpol} \cdot \left( {k + e} \right),} \\ \tau &= \frac{{tr_{len}}}{{l \cdot vpol}} \cdot \left( {k + e + m} \right) \end{array}$$

and is additionally determined by the relative length of the sequence that needs to be transcribed until RBP binding starts (τ_inh,1_) or ends (τ_inh,2_), or until inhibitor-independent commitment to skipping is possible (τ). Therefore, each delay increases with increasing total transcript length (tr_len_), given as the total number of nucleotides. Additionally, there are terms describing the proportion of the delay within the elongating transcript. For instance, τ_inh,1_ contains the term k/l, which equals the fraction of the sequence stretch before RBP inhibitor binding is possible (k) divided by the sum of all sequence stretches *l* = *k* + *e* + *m* + *n*. Likewise, the delays τ_inh,1_ and τ are scaled by (k + e)/l and (k + e + m)/l, respectively, where e is the sequence length of the RBP inhibitor binding window-of-opportunity and m is the duration of the elongation phase to the end of exon 3 after this window.

Our modeling study was motivated by our previously published experimental and theoretical analysis of a three-exon minigene that comprises exon 10-12 of the *ron* receptor tyrosine kinase gene^43^. Therefore, for all time delay simulations in Figs. 1 and 2, the modeled total transcript length was assumed to be *tr*_*len*_ = *300 nucleotides*, which falls into the range of the length of RON pre-mRNA segment between the end of exon 2 and the end of the transcript, i.e., the end of the third exon + ≈50 nts. The parameters k, e and l were chosen to mimick different RBP inhibitor binding positions within the transcript, and are given in Supplementary Table 1, with rate parameters provided in Supplementary Table 6.

In SBML format the time delays are realized as time triggered *Events*.

To validate our numerical simulations of the time delay model, we derived an analytical solution (Supplementary Table 1). As expected, we found an excellent agreement of numerical and analytical results in Figs. 1c and 2d.

The approach for calculating the analytical solution of the time delay model is based on the probability of commitment reaction towards inclusion. Under the assumption that the simulation starts with a value of one for the mRNA species (all others zero), one can calculate the probability of inclusion reaction for each of the four time phases *dt1, dt2, dt3, dt4* (Supplementary Figure 2), corresponding to the numerical integration intervals in Supplementary Table 1.

The first step is the calculation of *p1* representing the probability for inclusion within the first phase *dt1*, in which commitment to skipping is not possible (t < τ_inh,1_). Assuming a Poisson process, we can calculate the expected value $E_1 = A1 = ki \ast \tau _{inh,1}$, showing the amount of expected inclusion reactions within the phase 1 (*dt1* = *τ*_*inh,1*_). Assuming an exponential distribution, we get $p1 \in [0,1]$ as the value from cumulative distribution function

6 $$p_1 = 1 - e^{E_1}$$

The probability that the inclusion reaction will not take place in the first phase is $p_{rest1} = 1 - p_1$. The general formula for rest/remaining probability after the phase *i* is

7 $$p_{rest_i} = p_{rest_{i - 1}} - p_{react_i}$$

For *i* = 1, the value of $p_{rest_0}$ is 1, and $p_{react_1} = p_1$.

In the second phase (τ_inh,1_ < t < τ_inh,2_), there are two competing reactions, inclusion and RBP inhibitor-mediated commitment to skipping. The expected value for both reactions is $E_2 = A2 + A3 = \left( {k_{esc} + ki} \right) \ast (\tau _{inh,2} - \tau _{inh,1})$. The probability for one of the reaction will occur is $p_{react_2} = p_{rest_1} \ast (1 - e^{ - E_2})$. Or more generally expressed:

8 $$p_{react_i} = p_{rest_{i - 1}} \ast (1 - e^{ - E_i})$$

The probability for inclusion reaction results from:

9 $$p_2 = p_{react_2} \ast \frac{{A2}}{{A3 + A2}} = p_{react_2} \ast \frac{{ki}}{{ki + kesc}}$$

Consequently the rest probability for phase 3 is $p_{rest_3} = p_{rest_2} - p_{react_3}$.

For the two remaining phases (τ_inh,2_ < t < τ and t > τ), we can proceed in the same manner. It is important to note that $dt4 = \infty$. And in the forth phase there are two competing reactions, commitment to inclusion and skipping, which are handled similarly to the second phase.

Eventually we get the PSI-value as a sum of all absolute probabilities for inclusion *p*_*1*_*-p*_*4*_

10 $${{{\mathrm{PSI}}}} = p_{incl} = \mathop {\sum}\nolimits_{i = 1}^4 {p_i}$$

The analytical solution is a complex sum of exponential functions. For simplicity, this solution is not shown here, but it was used to generate the plots of the analytical solutions in Figs. 1c and 2d.

### Splicing commitment model – multistep implementation

To verify the time delay implementation, we also performed more conventional ODE simulations using an alternative model (Fig. 2b, top), in which pre-mRNA elongation is not simulated by a time delay, but by assuming a chain of consecutive first-order reactions (Fig. 2b), with parameters given in Supplementary Table 2. Specifically, we consider the transition between the transcript elongation states *P*_*i*_ → *P*_*i+1*_ and their RBP-inhibited counterparts *E*_*i*_ → *E*_*i+1*_ as reactions of first order with the reaction rate constant *k*_*elong*._ The ODE system describing the network topology in Fig. 2b (top) is given by

11 $$\begin{array}{ll} \frac{d}{{dt}}P_1 & = - P_1 \cdot \left( {k_{elong} + ki} \right)\hfill \\ \frac{d}{{dt}}Incl & = ki \cdot \left( {P_1 + P_2 + P_3 + P_4 + P_5 + P_6 + P_7 + P_8} \right) \hfill \\ \frac{d}{{dt}}Skip & = ks \cdot \left( {E_7 + E_8 + P_7 + P_8} \right) \hfill \\ \frac{d}{{dt}}P_2 & = P_1 \cdot k_{elong} - P_2 \cdot k_{elong} - P_2 \cdot ki \hfill \\ \frac{d}{{dt}}P_3 & = P_2 \cdot k_{elong} - P_3 \cdot k_{elong} - P_3 \cdot kesc - P_3 \cdot ki \hfill \\ \frac{d}{{dt}}P_4 & = P_3 \cdot k_{elong} - P_4 \cdot k_{elong} - P_4 \cdot kesc - P_4 \cdot ki \hfill \\ \frac{d}{{dt}}P_5 &= P_4 \cdot k_{elong} - P_5 \cdot k_{elong} - P_5 \cdot kesc - P_5 \cdot ki \hfill \\ \frac{d}{{dt}}P_6 &= P_5 \cdot k_{elong} - P_6 \cdot k_{elong} - P_6 \cdot ki \hfill \\ \frac{d}{{dt}}P_7 &= P_6 \cdot k_{elong} - P_7 \cdot k_{elong} - P_7 \cdot ki - P_7 \cdot ks \hfill \\ \frac{d}{{dt}}P_8 &= P_7 \cdot k_{elong} - P_8 \cdot ki - P_8 \cdot ks \hfill \\ \frac{d}{{dt}}E_3 &= - E_3 \cdot k_{elong} + P_3 \cdot kesc \hfill \\ \frac{d}{{dt}}E_4 &= E_3 \cdot k_{elong} - E_4 \cdot k_{elong} + P_4 \cdot kesc \hfill \\ \frac{d}{{dt}}E_5 &= E_4 \cdot k_{elong} - E_5 \cdot k_{elong} + P_5 \cdot kesc\hfill \\ \frac{d}{{dt}}E_6 &= k_{elong} \cdot \left( {E_5 - E_6} \right) \hfill \\ \frac{d}{{dt}}E_7 &= E_6 \cdot k_{elong} - E_7 \cdot k_{elong} - E_7 \cdot ks\hfill \\ \frac{d}{{dt}}E_8 &= E_7 \cdot k_{elong} - E_8 \cdot ks\hfill\end{array}$$

As for the time delay model, the initial state of all species is set to 0, with the exception of the initial unspliced pre-mRNA precursor *P*_*1*_, which is assumed to be 1. By integrating the ODE system using the function *odeint* from the phyton package *scipy (v. 1.3.1*, subpackage *integrate)*, we again calculate the probability for a pre-mRNA to result in skipping or inclusion isoforms.

Specifically, we perform time course simulations until *t* = *∞* (in practice t = 10^4^), check whether the values of P_i_ and E_i_ are close to zero 0 (all pre-mRNA spliced) and use the skipping and inclusion to calculate a PSI value (Eq. 4).

Notably, mRNAs are subject to constant synthesis and turnover in living cells, i.e., there is a permanent flux through the system. Importantly, the splicing outcomes (PSI values) we obtained using the numerical simulation procedure described above directly correspond to those of an extended system, in which the pre-mRNA is synthesized with a constant rate and the inclusion and skipping isoforms are subject to first-order degradation (not shown). This is due to the fact that all transcript elongation and splicing commitment reactions are irreversible in nature, i.e., the system in Eq. 11 functions as an irreversible decision module that has the same relative splicing outcome (PSI), irrespective of whether there is a permanent steady state flux or just a step-like pulse of mRNA synthesis, as we assumed here.

In Eq. 11, we assumed a total number of eight elongation steps (P1-P8). In the simulations in Fig. 1d, we varied the total number of elongation steps (l) and also considered a scenario with l = 80 (“many steps”), in addition to l = 8 (“few steps”). In this many steps model topology consisting of 80 ODEs, we proportionally increased, the number of steps in each of the four commitment regimes in Supplementary Figure 1 (topology parameters *k, e, m, n*). Specifically, we increased the number of steps in the initial inclusion-only regime from k = 2 in the few steps scenario to k = 20 with many steps. Similarly, the topology parameters e = 3, m = 1, n = 2 were increased to e = 30, m = 10, n = 20, respectively. Thus, the total number of steps is given by the sum of steps in the four regimes (*l* = *k* + *e* + *m* + *n*). Notably, the many steps simulation yielded qualitatively distinct results from the few steps scenario, and the time delay simulation agrees with the multistep model result for *l* = 80, whereas it differs from *l* = 8 (Fig. 1d). This is due to the fact that the multistep model with few steps gives rise to gradual transitions between the commitment regimes *k, e, m, n* in time, while for many steps these transition better resemble a delay, and thus reflect better our biological assumption that inhibitor-mediated or inhibitor-independent skipping is possible with high efficiency (i.e., in a step-like manner) as soon as the corresponding pre-mRNA sequences have been transcribed. In this sense, the multistep model with few steps is inaccurate, whereas the many steps simulation is a much better approximation of the time delay model. Both model topologies are provided as online SBML files.

The effective transcript elongation parameter (here k_elong_) in the model is not only determined by the RNA polymerase elongation speed (vpol), but also by the length of the gene in nucleotides (*tr*_*len*_) and - for the multistep model - by the number of elongation steps (l) that are considered in the model (see previous paragraph).

For all multi-step model simulations in Fig. 1d and those confirming the results in Fig. 2c (not shown), a fixed total transcript length of *tr*_*len*_ = *300* nucleotides was assumed. This is the length of pre-mRNA segment between the end of exon 2 (where the simulated transcript starts) and the transcript (i.e., third exon + ≈50 nts) end. The elongation rate constant k_elong_ in the model is proportional to the RNA polymerase elongation speed (vpol) and the total number of steps (l) and inversely proportional to the transcription length (*tr*_*len*_):

12 $$k_{elong} = \frac{{vpol \cdot l}}{{tr_{len}}}$$

In SBML format, this calculation is defined as *InitialAssignments*.

### Mechanistic exon definition model

To explicitly model binding of the inhibitory RBP to the pre-mRNA, we turned to mechanistic modeling (Figs. 3 and 4). Specifically, we considered RBP binding to the pre-mRNA, assumed that the RBP inhibits nearby splice sites and considered that introns may be retained if splicing becomes inefficient.

The mechanistic splicing model is schematically shown in Fig. 3a. It consists of two reaction sub-networks, one where the inhibitory RBP is not (yet) bound the pre-mRNA (left), and one where the RBP inhibitor is bound (right). In the following, we will initially describe the reactions in the absence of the RBP and will then discuss the implementation of RBP binding.

Splicing is catalyzed by the so-called spliceosome^6^. In the catalytic splicing cycle, pioneering spliceosomal subunits U1 and U2 recognize splice sites. Subsequently, further spliceosome subunits (U4-U6) are recruited which leads to assembly of a mature spliceosome complexes are introns and finally to the excision of introns. In our model, we focus on the key steps of the spliceosome cycle and describe only the initial binding of U1 and U2, followed by catalysis of the splicing reaction.

The description of initial splice site recognition is based on our previous work on exon definition (Fig. 3a and Enculescu et al.^46^). Specifically, we considered that all three exons may be “defined” by cooperative U1 and U2 binding across exons, as suggested by the literature on mammalian splicing^49,50,92^. In the model, the pre-mRNA is either unbound (P000, P000_inh) or one/multiple exons are recognized (all other states). For instance, both outer exons are defined in the states P101 and P111, whereas the middle exon is either undefined (P101) or defined (P111). Exon definition occurs in a combinatorial fashion and the binding reactions proceed irreversibly with the rate constants k_1_-k_3_ (Fig. 3a, left).

The spliceosome binding patterns impact on splicing outcomes. Specifically, splicing events can only happen if both flanking exons are defined. Therefore, we assume first-order splicing reactions (with the rate constant k_spl_) to inclusion (from state P111) if all exons are defined, or to skipping (from state P101) if all exons except the middle one are defined. Splicing can also be unproductive if the exons are not properly defined for inclusion or skipping (all other states), or if the splicing reactions do not occur in time in the states P101 and P111. Therefore, a first-order retention reaction is also considered in the model (rate constant k_ret_), but this only occurs post-transcriptionally, i.e., after transcription has been terminated.

As depicted in Fig. 3a (right), the inhibitory RBP modulates splicing outcomes by binding to all unspliced pre-mRNA states (e.g., P000 → P000_inh). For simplicity, we modeled all RBP binding steps as irreversible first-order reactions with the reaction rate constant *rbp*_*br*_. Subsequently, spliceosome binding occurs at reduced rates (k_1,inh_-k_3,inh_), but splicing (k_spl_) and retention (k_ret_) rates remain affected by RBP binding. Thus, the inhibitory RBP changes splicing outcomes by blocking initial spliceosome recruitment.

The ODE system describing the mechanistic model is given by

13 $$\begin{array}{ll} \frac{d}{{dt}}P_{000} &= - P_{000} \cdot \left( {k_1 + k_2 + k_3 + k_{ret} + rbp_{br}} \right)\\ \frac{d}{{dt}}P_{100} &= P_{000} \cdot k_1 - P_{100} \cdot k_2 - P_{100} \cdot k_3 - P_{100} \cdot k_{ret} - P_{100} \cdot rbp_{br}\\ \frac{d}{{dt}}P_{010} &= P_{000} \cdot k_2 - P_{010} \cdot k_1 - P_{010} \cdot k_3 - P_{010} \cdot k_{ret} - P_{010} \cdot rbp_{br}\\ \frac{d}{{dt}}P_{001} &= P_{000} \cdot k_3 - P_{001} \cdot k_1 - P_{001} \cdot k_2 - P_{001} \cdot k_{ret} - P_{001} \cdot rbp_{br}\\ \frac{d}{{dt}}P_{110} &= P_{010} \cdot k_1 + P_{100} \cdot k_2 - P_{110} \cdot k_3 - P_{110} \cdot k_{ret} - P_{110} \cdot rbp_{br}\\ \frac{d}{{dt}}P_{101} &= P_{001} \cdot k_1 + P_{100} \cdot k_3 - P_{101} \cdot k_2 - P_{101} \cdot k_{ret} - P_{101} \cdot k_{spls} - P_{101} \cdot rbp_{br}\\ \frac{d}{{dt}}P_{011} &= P_{001} \cdot k_2 + P_{010} \cdot k_3 - P_{011} \cdot k_1 - P_{011} \cdot k_{ret} - P_{011} \cdot rbp_{br} \\ \frac{d}{{dt}}P_{111} &= P_{011} \cdot k_1 + P_{101} \cdot k_2 + P_{110} \cdot k_3 - P_{111} \cdot k_{ret} - P_{111} \cdot k_{spli} - P_{111} \cdot rbp_{br} \\ \frac{d}{{dt}}P_{100inh} &= P_{000inh} \cdot k_{1inh} + P_{100} \cdot rbp_{br} - P_{100inh} \cdot k_{2inh} - P_{100inh} \cdot k_{3inh} - P_{100inh} \cdot k_{ret} \\ \frac{d}{{dt}}P_{000inh} &= P_{000} \cdot rbp_{br} - P_{000inh} \cdot k_{1inh} - P_{000inh} \cdot k_{2inh} - P_{000inh} \cdot k_{3inh} - P_{000inh} \cdot k_{ret}\\ \frac{d}{{dt}}P_{010inh} &= P_{000inh} \cdot k_{2inh} + P_{010} \cdot rbp_{br} - P_{010inh} \cdot k_{1inh} - P_{010inh} \cdot k_{3inh} - P_{010inh} \cdot k_{ret}\\ \frac{d}{{dt}}P_{001inh} &= P_{000inh} \cdot k_{3inh} + P_{001} \cdot rbp_{br} - P_{001inh} \cdot k_{1inh} - P_{001inh} \cdot k_{2inh} - P_{001inh} \cdot k_{ret} \\ \frac{d}{{dt}}P_{110inh} &= P_{010inh} \cdot k_{1inh} + P_{100inh} \cdot k_{2inh} + P_{110} \cdot rbp_{br} - P_{110inh} \cdot k_{3inh} - P_{110inh} \cdot k_{ret}\\ \frac{d}{{dt}}P_{101inh} &= P_{001inh} \cdot k_{1inh} + P_{100inh} \cdot k_{3inh} + P_{101} \cdot rbp_{br} - P_{101inh} \cdot k_{2inh} - P_{101inh} \cdot k_{ret} - P_{101inh}k_{spls}\\ \frac{d}{{dt}}P_{011inh} &= P_{001inh} \cdot k_{2inh} + P_{010inh} \cdot k_{3inh} + P_{011} \cdot rbp_{br} - P_{011inh} \cdot k_{1inh} - P_{011inh} \cdot k_{ret}\\ \frac{d}{{dt}}P_{111inh} &= P_{011inh} \cdot k_{1inh} + P_{101inh} \cdot k_{2inh} + P_{110inh} \cdot k_{3inh} + P_{111} \cdot rbp_{br} - P_{111inh} \cdot k_{ret} - P_{111inh}k_{spli}\\\frac{d}{{dt}}ret &= k_{ret}\left( P_{000} + P_{000inh} + P_{001} + P_{001inh} + P_{010} + P_{010inh}+P_{011} + P_{011inh} + P_{100}\right.\\ &\left.\qquad +\, P_{100inh} + \,P_{101} + P_{101inh} + P_{110} + P_{110inh} + P_{111} + P_{111inh}\right)\\ \frac{d}{{dt}}Incl &= k_{spli} \cdot \left( {P_{111} + P_{111inh}} \right) \\ \frac{d}{{dt}}Skip &= k_{spls} \cdot \left(P_{101} + P_{101inh}\right) \end{array}$$

Numerical integration of the ODE system in Eq. 13 with time-invariant rate constants yields simulations of post-transcriptional splicing in the presence of RBP binding. Co-transcriptional splicing can be simulated by implementing time-dependent changes in the reaction rate constants, thereby mimicking changes in binding site availability during transcript elongation. As for the splicing commitment model, we implemented this behavior of co-transcriptional splicing using time delays, but this time started our simulations (t = 0) at the time of transcript initiation (not when exon 2 transcription has been finished).

Co-transcriptional spliceosome binding was considered in the model by assuming that an exon can be defined only after its synthesis is complete. Hence, the rate constants k_1_-k_3_ and k_1,inh_-k_3,inh_ change over time and increase in a step-like manner with delays (τ_1_-τ_3_ in Supplementary Table 2) that reflect the relative positions of exons within the pre-mRNA (Fig. 3c). In this work, we used the structure of the RON mini-gene^43^, and hence assumed that exon 1, 2 and 3 end (i.e., k_1_-k_3_ increase) at 210, 443 and 690 nucleotides after transcript initiation. Using these sequence positions (u1_ex1_-u1_ex3_ in Supplementary Table 1) and the RNA polymerase speed (vpol), given in nucleotide per second, we calculate the delays τ_1_-τ_3_ (see Supplementary Table 3).

In the model, we did not assume a time-dependence of splicing reaction rate constant (k_spl_), but assumed that intron retention can only take place after transcription is terminated (i.e., 700 nucleotides after transcript initiation). The transcription termination position *gene*_*len*_ = *700* together with the RNA polymerase speed is used to calculate the corresponding delay τ_6_ (see Supplementary Table 3). By this time dependence of intron retention, we reflect in the model that transcripts released from RNA polymerase may eventually exit the nucleus, where they can no longer be spliced.

For the inhibitory RBP, it was implemented that the binding reaction (*rbp*_*br*_) can only occur once the RBP motif has been transcribed and additionally assumed that RNA polymerase deposits the RBP on the pre-mRNA co-transcriptionally. Hence, RBP binding is modeled as a short window-of-opportunity, described by two delays τ_4_ and τ_5_ (see Supplementary Table 3). τ_4_ reflects the sequence position of the RBP binding motif, normalized by the polymerase speed (Supplementary Table 3), whereas τ_6_ marks the sequence position where elongating polymerase is no longer able to deposit the bound RBP on its sequence motif. Hence, a deposition range (pol_range_) is assumed in the model (Supplementary Table 1) which represents the molecular flexibility of the elongating enzyme and the size of the RBP that needs to be deposited.

Taken together, the model contains six time delays, whose values used in each figure of the paper are summarized in Supplementary Table 1, alongside with the other parameters of the model specified in Supplementary Table 6. Depending on the position of the RBP binding motif, the temporal order of the delays τ_1_-τ_6_ may change. To ensure correct order, the calculated delays are sorted before the integration of the ODE system, and the integration is then done in seven time intervals, in a manner similar to the integrations of the ODE system of time delay commitment models. The simulation starts at time t = 0 (transcription initiation) and we set all species to zero, except for the P000, which is set to 1. Then the first phase will be integrated until first τ. All subsequent phases uses the end species vector from the previous time phase as initial state. Then, alternative splicing is quantified using the PSI metric (Eq. 4).

In our model, the RBP bound to its sequence motif inhibits the recognition of nearby splice sites. Since we assume that both splice sites of an exon are bound cooperatively, the RBP reduces the exon definition reactions in our model. Thus, the parameters k_1,inh_-k_3,inh_ may be smaller than their counterparts in the absence of RBP binding (k_1_-k_3_). Importantly, this inhibition effect occurs only locally around the site of RBP binding (Fig. 3b). For simplicity, we initially assumed a bell-shaped inhibition profile, in which the reduction of k_1,inh_-k_3,inh_ relative to k_1_-k_3_ occurs only within ~100 bp around the RBP binding site.

The values of k_1,inh_-k_3,inh_ (k_x,inh_) are described by the following inhibition function (that is also depicted in Fig. 3b).

14 $$k_{x,inh} = k_x \ast \left( {1 - inhFunc\left( {5^\prime SS} \right)} \right) \ast (1 - inhFunc\left( {3^\prime SS} \right))$$

Here, 5´SS and 3´SS reflect the relative distance of the upstream and downstream splice sites of an exon to the RBP binding site in nucleotides. Due to the restricted spatial range of RBP-mediated inhibition in our simulations, we neglected long-range RBP interactions with more distal splice sites. Before each simulation, the values k_1,inh_-k_3,inh_ are calculated using the model parameters and in the sbml files this is done by *InitialAssignments*.

The inhibition function (*inhFunc* in Eq. 14) is a parameterized piecewise-defined function with Hill-type terms

15 $$InhFunc_{l,r,p}\left( x \right) = \left\{ {\begin{array}{*{20}{c}} {\frac{1}{{\left( { - \frac{x}{l}} \right)^p + 1}}for\;x \,<\, 0} \\ {\frac{1}{{\left( {\frac{x}{r}} \right)^p + 1}}otherwise} \end{array}} \right.$$

Here, the parameters *l, r*, and *p* determine the range and the shape of the inhibition function

*l* – upstream range in nucleotides

*r* – downstream range in nucleotides

*p –* hill-coefficient like parameter determining the shape /steepness of the function

*x –* distance from RBP binding site in nucleotides

The values chosen for the simulations in Figs. 3 and 4 are summarized in Supplementary Table 5.

### Stochastic simulations

To quantify uncertainties in splicing outcomes, we performed stochastic simulations using our co-transcriptional splicing models in previous sections (Splicing commitment model - time delay implementation and Mechanistic exon definition model). The simulation results were generated using the Gillespie algorithm^63^. Since all reaction steps in our splicing models are of first order, the kinetic parameter values in the ODE models can directly be used as reaction probabilities in the Gillespie simulations.

#### Splicing commitment models

The simulations in Fig. 6a and c were performed using the time delay model from Fig. 2c (bottom), with the reaction probability values summarized in Supplementary Table 6. For Fig. 6b we used all 3 models from Fig. 3c plus the time delay model from Fig. 1d. During the Gillespie simulations, the reaction probabilities were assumed to increase in a step-like manner with time delays corresponding to those described in Section Splicing commitment model - time delay implementation. At the initial time point all species except for the mRNA were set to zero. In Fig. 6a and c, the initial state of mRNA species was set to 100 molecules, whereas it was varied between 10 and 1000 molecules in Fig. 6b (see legend). Figure 6b contains simulations with various PSI outcomes. As in Figs. 1 and 2, we generated these PSI by varying the RNA polymerase elongation speed vpol between 1 and 1000 nucleotides per second. It was done for each of 4 models.

In Fig. 6a and c, we show time courses for 100 realizations, whereas Fig. 6b contains final splicing outcomes (at t = 1000) for 5000 realizations. The PSI metric was calculated for each individual realization (cell), and the PSI mean and standard deviation were calculated based on the PSI distributions across 5000 cells.

For comparison of stochastic splicing outcomes with a binomial model (thin lines in Fig. 5b), we sampled binomial distributions using the *stats.binom.std* command from the python package *scipy* (v. 1.3.1). Here, we varied the number and probability of sampled events to mimick varying molecule counts and varying mean(PSI) values, respectively. The standard deviation of the obtained binomial distribution was plotted as the std(PSI) (thin lines in Fig. 5b).

#### Mechanistic exon definition model

The Gillespie simulations of the mechanistic model (Fig. 6d-f) were performed using the reaction probabilities summarized in Supplementary Table 5 and by setting all initial molecule counts to zero except for the precursor P000. In Fig. 6d and f, the initial state of *P000* was set to 100, whereas it was varied between 10 and 40 molecules in Fig. 6e. In Fig. 6e, the final PSI value of the time courses was recorded at t = 1000, and variations in PSI were introduced by changing the RBP binding site position between 220 and 280 nts downstream of the transcription start site. The stochastic simulation results therefore directly correspond to the thick blue simulation outcome of the deterministic model in Fig. 4b (top panel) in the range of 220-280 nucleotides.

In Fig. 6d and f, we show time courses for 100 realizations, whereas Fig. 6e contains final splicing outcomes (at t = 1000) for 5000 realizations. The PSI metric was calculated for each individual realization (cell), and the PSI mean and standard deviation were calculated based on the PSI distributions across 5000 cells.

In the mechanistic model, the comparison of the stochastic simulations (Fig. 6e) could not be done based on the noise-mean relationship as for the splicing commitment model (Fig. 6b). The reason is that the total molecule count in the mechanistic model (Fig. 6e) does not directly correspond to the initial amount of the P000 species, because intron retention occurs as a third (noisy) splicing outcome, in addition to skipping and inclusion. Therefore, for each realization of the mechanistic model, mean and standard deviation of PSI are calculated for different absolute counts of the relevant molecules (sum of skipping and inclusion). Consequently, noise-mean-relationships at defined absolute molecule counts as in Fig. 6b cannot be obtained for the mechanistic exon definition model. Thus, in Fig. 5e we calculated the correlation of the binomial and stochastic noise (std(PSI)) by assuming the same mean(PSI) and absolute molecule count (sum of skipping and inclusion in the mechanistic case) for both models. Hence, if fluctuations in the total amount of skipping and inclusion are corrected for, the mechanistic exon definition model perfectly corresponds to the binomial case.

#### Bimodality

To establish bimodality in alternative splicing outcomes, we implemented an extended version of the stochastic splicing commitment model (subsection i, Fig. 5a-c). The extended model contains positive feedback regulation and stochastic promoter switching between transcriptionally active (Prom_on_) and inactive (Prom_off_) states as additional mechanisms of regulation (Fig. 7a, b, e, f).

Positive feedback is implemented by assuming that the protein product of the skipping isoform serves as an RBP that binds to its own pre-mRNA precursor and enhances the production of the skipping isoform. A basal level of the skipping isoform is committed to at the same rate as the inclusion isoform, albeit only once the transcript is nearly fully synthesized, therefore minimizing the amount of skipping isoform generated in the absence of feedback amplification due to the much later opportunity to commit to skipping. We neglected the molecular details of RBP protein biosynthesis and binding to pre-mRNA, and implemented positive feedback (+ve in Fig. 7a) using a Hill-type equation that adds an additional pathway to skipping commitment, with a propensity that is 0 in the absence of skipping isoform, but otherwise specified as:

16 $$FeedbackPropensity = \frac{{Fb_S}}{{1 + \left( {\frac{K}{{Skip}}} \right)^N}}$$

The parameters Fb_s_, K, and N determine the magnitude, threshold, and sensitivity of the feedback respectively.

As an additional mechanism in the extended model, we considered stochastic promoter switching between transcriptionally active and inactive states (Prom_on_ and Prom_off_ in Fig. 7a), e.g., due to formation and dissociation of transcription factor complexes. This model extension, known as the random telegraph model^93,94^, establishes transcriptional bursts in mRNA expression time courses. During a burst, a high amount of transcripts is generated, whereas no transcription occurs between bursts. This, combined with different transcript lifetimes of inclusion and skipping mRNAs, can give rise to bimodal behavior in PSI in the absence of feedback: suppose that inclusion is the isoform that is predominantly generated during a burst (PSI > 0.5). After the burst, both inclusion and skipping isoforms will decay (until the next burst starts again). If inclusion is much less stable than skipping, the PSI will quickly drop after the burst, eventually reaching PSI = 0 during most of the waiting time till the next burst. To account for this we specified the degradation rates for the inclusion and skipping isoforms individually.

Implementation of bimodal variants of the model utilized the model depicted in Fig. 7, with (i) feedback amplification being set to zero in the stochastic bursting analysis (Fig. 7b and c); (ii) stochastic bursting being eliminated when focusing on the effects of positive feedback (Fig. 7d and e); (iii) both mechanisms considered simultaneously in Fig. 7 f and g. All stochastic simulations were performed using the Gillespie algorithm with a total of 10,000 time points. The histograms and time courses in Fig. 7 show fluctuations after the simulation reached steady state. For comparison, the time courses in Fig. 7 also contain simulations of the corresponding ODE system, with an initial condition of 1 Prom_on_ and 0 pre-existing transcripts.

Parameter values used for the simulations in Fig. 7 are provided in Supplementary Table 4. These parameter values were obtained by scanning the parameter space for the occurrence of bimodal behavior: For bimodality from transcriptional bursting and variable degradation (Fig. 7b and c), parameters were chosen based on scanning of the parameter values of k_i_, k_s_, commitment to inclusion and skipping respectively, and d_i_, and d_s_, representing degradation of the inclusion and skipping isoforms. Starting from equal values, we obtained bimodal behavior by simultaneously increasing k_i_ and d_i_, and decreasing k_s_ and d_s_. For the parameter scanning in the positive feedback regulation scenario (Fig. 7d and e), the coefficient N was chosen as a random value greater than 3. K was chosen to equal a high value of the skipping isoform that was rarely achieved in simulations without feedback, ensuring rare activation of the feedback loop. Finally, the parameter Fb_s_ was determined by scanning for values that permitted bimodality.

#### Comparison of model simulations to data from Coulon *et al*

Our co-transcriptional splicing models aim to provide a generic framework to quantitatively analyze how the RNA’s fate is determined by the coordination of fundamental enzymatic reactions required in RNA synthesis, such as transcription initiation, elongation and splicing. How the models behave confronting the experimental data is invaluable to test the validity of our theory. Yet measuring simultaneously multiple enzymatic reactions during RNA synthesis is experimentally challenging, especially in single cells. However, Coulon et al.^59^ obtained such type of data in single cells in great temporal and spatial details. We therefore use their data to further challenge our models.

Coulon et al. performed dual-color labeling of single transcripts of a human β-globin reporter to assess whether single-intron splicing displayed dependencies on the processes of transcript elongation, and transcript end processing and release: specifically, they simultaneously labeled an intron (removed by splicing) and terminal exon (not removed by splicing) using two different fluorophores. Through measuring the co-localization and concentration of the two fluorophores, they were able to monitor transcript elongation, intron splicing and transcript release from chromatin at the single transcript level, allowing them to determine which fraction of a transcript splicing occurs co- or post-transcriptionally, and which processes were dependent on the completion of the others. Individual kinetic parameters of these processes (e.g., the elongation rate, splicing time, and the rate of transcript release from chromatin) were inferred from the data using a quantitative stochastic modeling approach.

We assessed whether our model of co-transcriptional splicing is consistent with the data reported by Coulon et al. Notably, the stochastic model which these authors used to quantitatively describe their data is similar to ours, as they also allow splicing to happen with a delay during transcript elongation, i.e., after the required cis-regulatory splicing elements (splice sites) have been synthesized. Moreover, in the model variant that is most consistent with the data, the authors assumed that transcript elongation and splicing are independent processes, i.e., perturbing splicing does not affect elongation and vice versa. This already suggests that our model may be suitable to quantitatively describe their data. However, there are also important differences between our model and the one reported by Coulon et al.: (I) their model describes a single intron flanked by two exons, whereas we describe a complete three-exon minigene including two introns flanking an alternative exon; (ii) we assume an exon definition mechanisms of splicing, whereas they assumed an intron definition mechanism; (iii) in their description, splicing is a multistep process (the corresponding splicing time has a peaked distribution), while we assumed a single-rate limiting step for splicing (exponentially distributed splicing time). Given these differences in the two models, we asked whether our model is able to quantitatively describe co-transcriptional splicing kinetics reported in Coulon et al.

To this end, we employed kinetic parameters inferred by Coulon for five experimental conditions, plugged them into our model (as described further below), performed stochastic simulations of co-transcriptional splicing, and compared them to the percentage of transcripts spliced before release from chromatin, which Coulon et al. had observed directly using autocorrelation analysis of the data. Besides for the wildtype conditions, this analysis was performed for experimental perturbations reported in Coulon et al., in which the rates of transcript elongation (campothecin, CPT) or intron splicing (spliceostatin A, SSA; expression of the U2AF1 mutant S34F) were altered independently of one another (described further below).

The stochastic model we used for comparison to the Coulon et al. data is depicted in Supplementary Figure 3. As a solid criterion to compare with the stochastic simulation performed later, we derived an analytical solution for the percentage of co-transcriptionally spliced transcripts. It provides an overview of the parameters, that permits usage of the reported error bounds to simulate additional datapoints. In addition, as we utilize the abstraction of elongation steps in our model, it was deemed prudent to determine how the number of steps may impact the results of the model:

17 $$\% SplicedPrerelease = 100 \times \left[ {1 - \left( {\frac{{k_{elong}}}{{k_i + k_{elong}}}} \right)^{no.steps} \times \frac{{tr}}{{k_i + tr}}} \right]$$

The term k_elong_/(k_i_ + k_elong_) reflects that each elongation step of the unspliced transcript (P_i_) is characterized by competition between (i) splicing commitment and catalysis (k_i_) and (ii) further elongation into the next unspliced elongation step (k_elong_). Here, the splicing reaction is assumed to occur right after commitment, so the k_elong_/(k_i_ + _kelong_)-term reflects the decision to either splice co-transcriptionally in this elongation step, or to proceed into the next unspliced elongation state (P_i+1_ in Supplementary Figure 3a). The overall probability of arriving in the last unspliced elongation step (P_8_ in Supplementary Figure 3a) is then the k_elong_/(k_i_ + k_elong_)-term raised to the power of the total number of elongation steps (no. steps). As elongation comes to an end, there is competition between transcript release (tr) and the commitment and splicing of the intron (k_i_), implying multiplication with an additional tr/(k_i_ + tr)-term. The resulting product describes the probability of a transcript to be released from chromatin before splicing, and the sought after % co-transcriptional splicing is given by 1 minus this product, multiplied by a factor of 100. In order to independently validate the analytical solution in Eq. 17., the stochastic model was also implemented numerically using the Gillespie Algorithm.

The kinetic parameters describing transcript elongation, intron splicing and transcript release from chromatin inferred by Coulon et al. using stochastic model fitting are summarized in Table 1 of their paper. As all reactions in our model follow first-order kinetics, we are able to directly convert rate parameters obtained from Table 1 of Coulon et al. into reaction propensities for simulation using the Gillespie Algorithm, or for use in the analytical solution (Eq. 17).

The elongation rate k_elong_ in our model was obtained via multiplying the polymerase speed (reported in Table 1 of Coulon et al.) by the number of elongation steps in our model divided by the length of the experimentally characterized β-globin reporter, providing the rate for a single elongation step in our model:

18 $$K_{elong} = ElongationRate = PolSpeed \times \frac{{no.steps}}{{TranscriptLength}}$$

The transcript release rate in our model from is calculated from the inverse of the mean 3ʹ end dwell time reported in Coulon et al.

The co-transcriptional splicing rate in our model was calculated based on the percentage of transcripts spliced co-transcriptionally, as reported by Coulon et al., divided by the above reported polymerase speed and release time:

19 $$K_i = PreReleaseSpliceRate = \frac{{PreReleaseSplice\% }}{{\frac{L}{{PolSpeed}} + 3^\prime EndDwellTime}}$$

where *L* is the length between the 3ʹ splice site of the reporter construct to the end of the poly-a tail, whose value, 2353 nucleotides, was taken from Fig. 1 in the Coulon et al. paper.

From Coulon et al. we obtain these parameters from 5 different experimental conditions reported in Table 1: a WT control, treatment with spliceostatin (SSA + ) to inhibit splicing, treatment with camptothecin (CPT), a topoisomerase inhibitor that reduces the polymerase elongation speed, transfection with a WT copy of the splicing factor U2AF1, and transfection with a U2AF1 allele containing a missense mutation S34F, that reduces both the splicing rate and the transcript release rate. For each datapoint, error bounds are reported, and these were used to create minimal and maximal values for the pre-release splicing percentage. We used the minimal value of the pre-release spliced percentage for the lower bounds, along with the maximum polymerase speed and minimum 3´ end dwell time, and vice versa for the maximum value of the pre-release splicing percentage. These sets of parameters were then input into the analytical solution (Eq. 17) and stochastic simulations implemented as described in the previous sections, with the model scheme depicted in Supplementary Figure 3 and an initial state of 10,000 units of P1.

We can observe in the graph of Supplementary Figure 3 that these solutions can faithfully reconstruct the experimentally reported pre-release splicing percentage at low values, with slightly decreasing accuracy at higher values. We conclude that our model displays similar accuracy to pre-existing models supported by experimental data, whilst providing substantial extensibility, as demonstrated by the use of similar model topologies throughout this paper to demonstrate varied regulatory aspects of alternative splicing.

## Supplementary information

Supplementary Information

## Data Availability

All model source code and relevant data are available from the authors upon request.
