# Supplementary material for: Position-dependent effects of RNA-binding proteins in the context of co-transcriptional splicing
Source: NPJ Syst Biol Appl. 2023 Jan 18;9:1. doi: 10.1038/s41540-022-00264-3 (PMC9849329; doi:10.1038/s41540-022-00264-3)
Supplement: Supplementary file 1 — Supplementary Information [file 41540_2022_264_MOESM1_ESM.pdf]

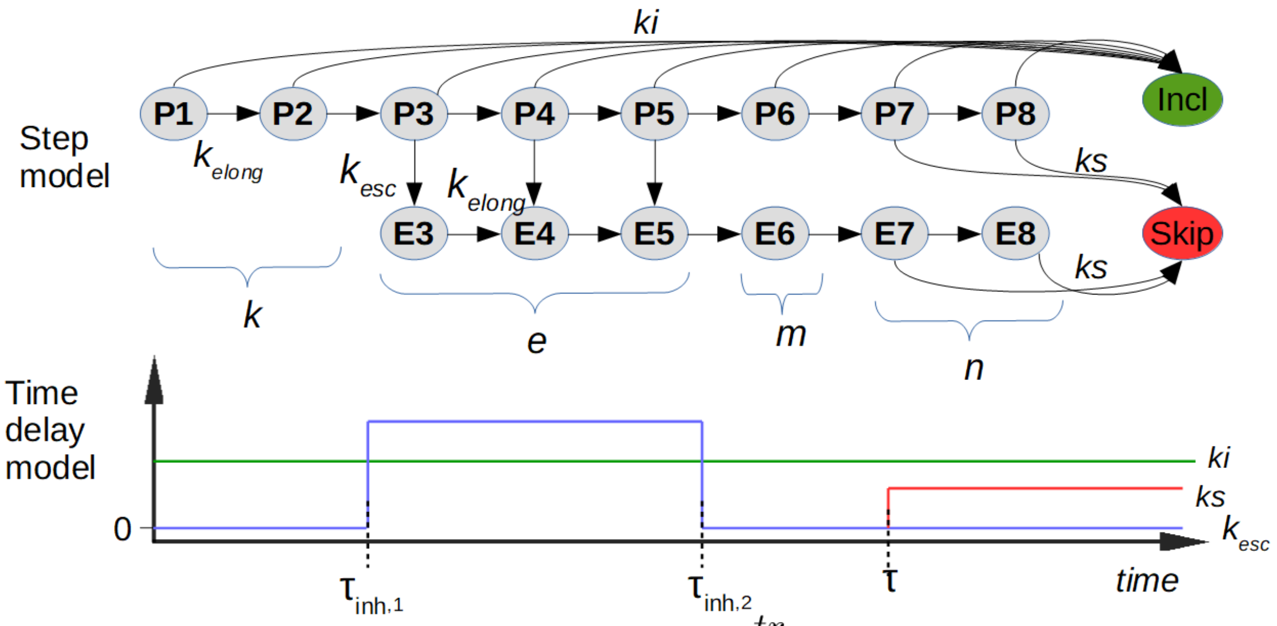

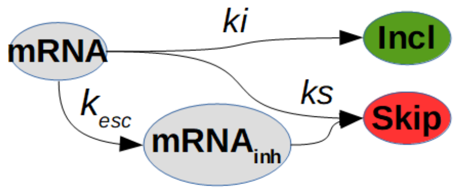

$$\tau_{inh,1} = \frac{tr_{len}}{l \cdot vpol} \cdot k,$$

$$\tau_{inh,2} = \frac{tr_{len}}{l \cdot vpol} \cdot (k + e)\,,$$

$$\tau = \frac{tr_{len}}{l \cdot vpol} \cdot (k + e + m)$$

Supplementary Figure 1. Relationship between gene structure, polymerase speed and time delay in the time delay and multistep models of co-transcriptional splicing.

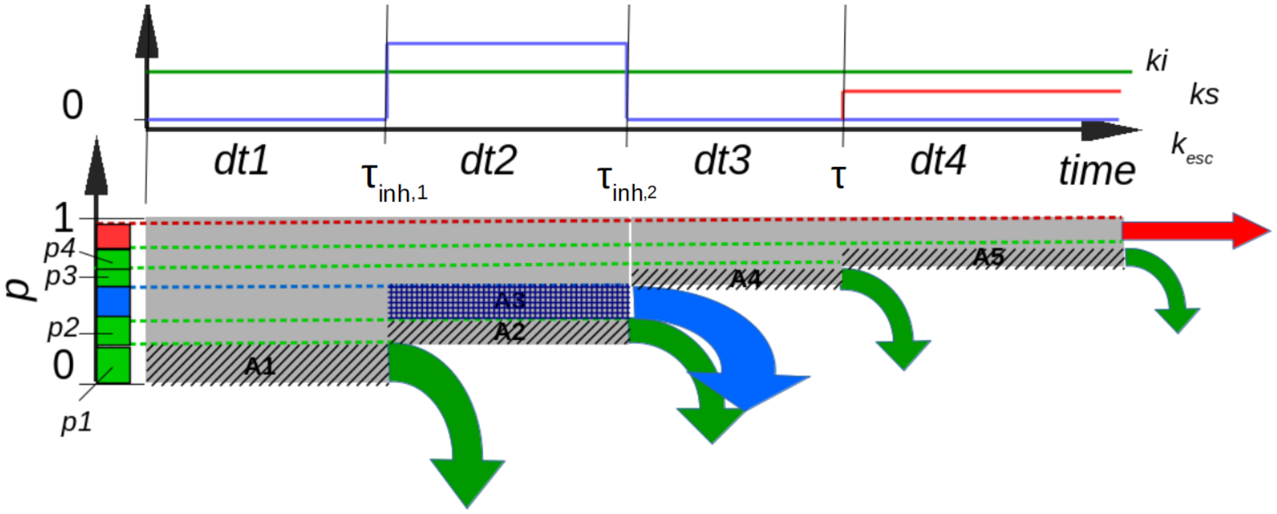

Supplementary Figure 2. Derivation of an analytical solution for the time delay model. On the top, time-dependent changes of the commitment reaction rates towards inclusion (ki) and skipping (ks, kesc) are indicated. The bottom schematically indicates the probability of commitment and the abundance of committed species over time. Initially, only commitment to inclusion is possible (bottom green arrow), then inclusion and inhibitor-mediated commitment occurs (red and blue arrows, middle), followed by inclusion only (green arrow) and then by a mixture of skipping and inclusion (red and green arrows). The analytical approach is based on this model (bottom). p1-p4 are absolute probabilities for inclusion reaction in the corresponding phases 1-4.

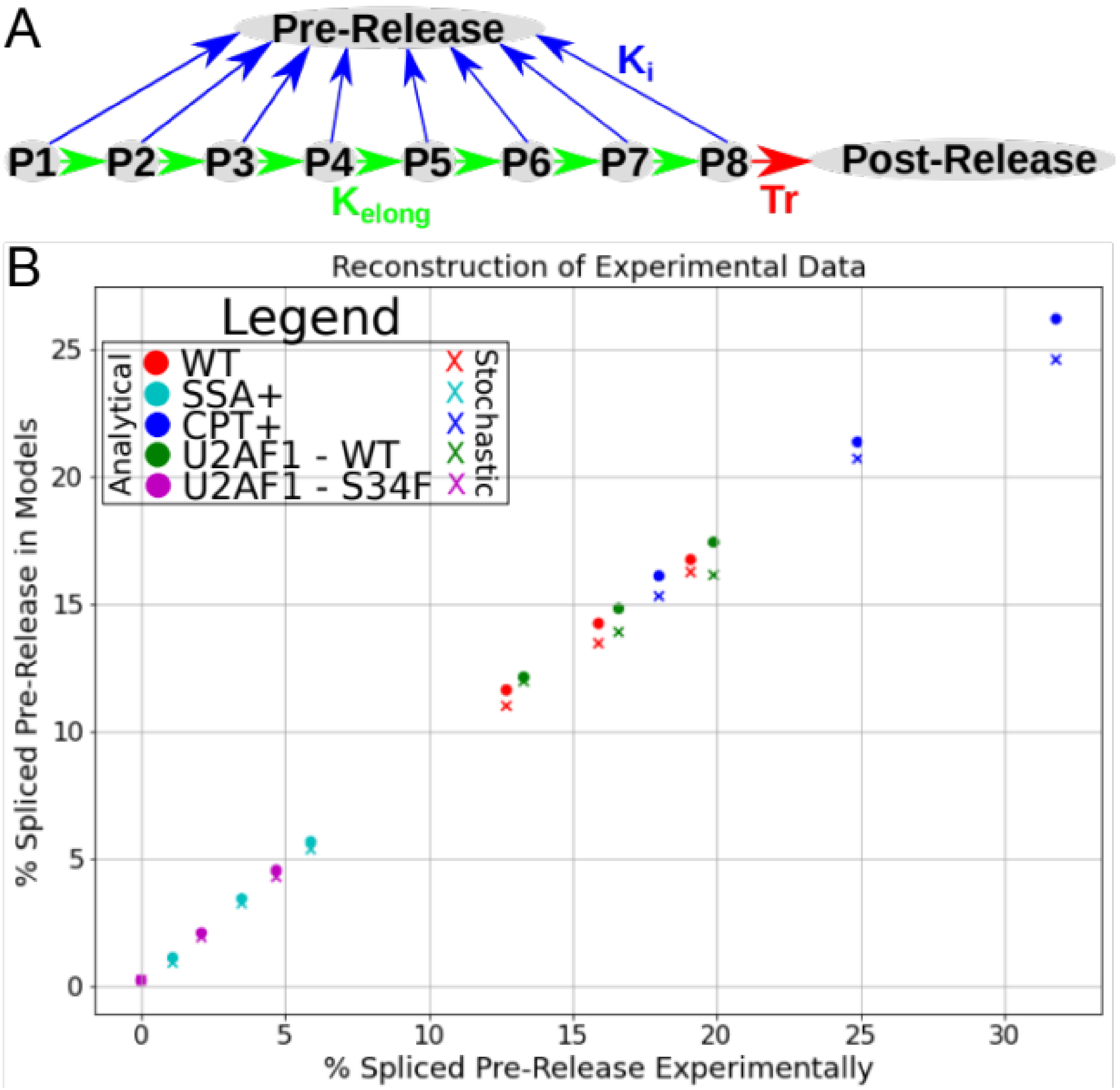

Supplementary Figure 3. Step-wise co-transcriptional splicing models reproduce experimental data observed in Coulon et al. (A) Model scheme. The rate K_{elong} is a parameterisation of the RNA polymerase speed, Ki of the commitment and catalysis of splicing, and Tr representing 3´ end processing and transcript release. The X-axis corresponds to the column “Pre-release Fraction (%)” in Table 1 of Coulon et al., with a datapoint, minimum, and maximum value for each row plotted. Color code represent different experimental conditions. Dots and crosses stand for analytical and stochastic simulation results, respectively. Parameters for the minimum and maximum values were chosen as outlined in Methods.

Supplementary Table 1: Four consecutive integration intervals in the presence of an RBP inhibitor that establishes a widow-of-opportunity for commitment to skipping (between τinh,1 and τinh,2) before exon 3 has been fully transcribed.

Time phase	constants	Effective ODEs
$0 - \tau_{inh,1}$	***ki = ki*** $\boldsymbol{ki = ki_{model}}$<br>*ks = 0*<br>*kesc = 0*	$\frac{d}{dt}mRNA = -mRNA \cdot ki$<br>$\frac{d}{dt}Incl = ki \cdot mRNA$
$\tau_{inh,1} - \tau_{inh,2}$	$\boldsymbol{ki = ki_{model}}$<br>*ks = 0*<br>$\boldsymbol{kesc = kesc_{model}}$	$\frac{d}{dt}mRNA = -mRNA \cdot (kesc + ki)$<br>$\frac{d}{dt}Incl = ki \cdot mRNA$<br>$\frac{d}{dt}mRNAinh = kesc \cdot mRNA$
$\tau_{inh,2} - \tau$	$\boldsymbol{ki = ki_{model}}$<br>*ks = 0*<br>*kesc = 0*	$\frac{d}{dt}mRNA = -mRNA \cdot ki$<br>$\frac{d}{dt}Incl = ki \cdot mRNA$
$\tau - \infty$	$\boldsymbol{ki = ki_{model}}$<br>$\boldsymbol{ks = ks_{model}}$<br>*kesc = 0*	$\frac{d}{dt}mRNA = -mRNA \cdot (ki + ks)$<br>$\frac{d}{dt}Incl = ki \cdot mRNA$<br>$\frac{d}{dt}Skip = ks \cdot (mRNA + mRNAinh)$<br>$\frac{d}{dt}mRNAinh = -ks \cdot mRNAinh$

Supplementary Table 2: Parameters for step-wise models. Vpol corresponds to RNA polymerase, but is converted into k_elong based on the formula vpol*l/tr_len to represent the transition rate between two steps. The number of steps is given by l, with tr_len giving the actual length in nts of the transcript. The parameters ki, ks, and kesc represent the rates of commitment to inclusion, skipping, and RBP-inhibition of inclusion respectively in steps where these reactions are possible. The step-wise model in figure 2c iii is not presented graphically, with parameters provided here to demonstrate the conversion between the parameters provided in Supplementary Table 6 for the time-delay model and the step-wise model.

Parameter	Figure 1f – Few Steps	Figure 1f – Many Steps	Figure 2c iii
Vpol	50	50	50
l	8	80	8
tr_len	300	300	300
k_elong	1.3333	13.3333	1.3333
ki	0.05	0.05	0.1
ks	0.5	0.5	0.02
kesc	0	0	0.5

Supplementary Table 3: Time delays in the mechanistic exon definition model and their calculation based on sequence position and RNA polymerase speed.

Time delay	Term	Description
τ1	$u1_{ex1}/vpol$	End of exon 1: k1 and $k1_{inh}$ on
τ2	$u1_{ex2}/vpol$	End of exon 2: k2 and $k2_{inh}$ on
τ3	$u1_{ex3}/vpol$	End of exon 3: k3 and $k3_{inh}$ on
τ4	$rbp_{pos}/vpol$	RBP binding side transcribed: rbp_{br} on
τ5	$\frac{rbp_{pos}+pol_{range}}{vpol}$	Pol II passes RBP binding site : rbp_{br} off<br>τ4 and τ5 constitute the window of opportunity for RBP binding
τ6	$gene_{len}/vpol$	Gene transcription end. Retention can take place: k_{ret} on

Supplementary Table 4: Parameter values derived from Coulon et al. for use in the validation presented in Supplementary Figure 3. Recurring values for Vpol are displayed to 3 significant figures, values for release and splicing rate are displayed to 1 significant figure.

Datapoint	% Spliced pre-release	Vpol (nt/s)	Release rate (s^{-1})	Splicing rate (s^{-1})
Control – Avg	15.9	43.333	0.009	0.001
Control – Min	12.7	46	0.009	0.0008
Control – Max	19.1	40.666	0.008	0.001
SSA+ – Avg	3.5	40.166	0.008	0.0002
SSA+ – Min	1.1	44.5	0.008	0.00006
SSA+ – Max	5.9	35.833	0.008	0.0003
CPT+ – Avg	24.9	24	0.009	0.001
CPT+ – Min	18	25.5	0.01	0.0009
CPT+ – Max	31.8	22.5	0.008	0.001
U2AF1 WT – Avg	16.6	37.333	0.008	0.0009
U2AF1 WT – Min	13.3	41.833	0.009	0.0008
U2AF1 WT – Max	19.9	32.833	0.008	0.001
U2AF1 S34F – Avg	2.1	44	0.006	0.00009
U2AF1 S34F – Min	0	45.833	0.006	$1e^{-5}$*
U2AF1 S34F – Max	4.7	22.5	0.005	0.0002

*Splicing rate for U2AF1 S34F minimal value is, in fact, 0, however to avoid numerical difficulties it is instead set to be at least one order of magnitude smaller than all other splicing rates, but greater than 0.

Supplementary Table 5: Parameter values for the mechanistic exon definition models.Vpol represents polymerase speed in nt/s, with gene_len representing the length of the gene in nts. The values u2/u1_exN represent the 3′ and 5′ splice sites of exon N respectively, which once synthesised result in the the exon defintion rates kN_t, and kN_inh_t when the inhibitor is bound, being set in the model. rbp_pos specifies the position at which the inhibitor may bind with the rate rbp_br_t, and pol_range specifies the length after rbp_pos that binding stops at. rbp_e_up, rbp_e_down, rbp_h_c, and rbp_inh all specify how the inhibitor effects splice site strengths once bound, as detailed in equation 15. k_spl_i, k_spl_s, and k_ret_t respectively mark the splicing rate of transcripts that meet the requirement for inclusion, skipping, and retention isoforms to be generated.

Parameter	Fig 3d i	Fig 3d ii	Fig 3d iii	Fig 3d iv	Fig 4 abc	Fig 4d	Fig 6 def
vpol	50	50	50	50	50	50	50
gene_len	700	700	700	700	700	700	700
u2_ex1	50	50	50	50	50	50	50
u1_ex1	210	210	210	210	210	210	210
u2_ex2	300	300	300	300	300	300	300
u1_ex2	443	443	443	443	443	443	443
u2_ex3	520	520	520	520	520	520	520
u1_ex3	690	690	690	690	690	690	690
k1_t	10	10	10	10	1	1	1
k2_t	0.2	0.1	0.2	0.2	0.1	0.1	0.1
k3_t	1	1	0.02	0.02	1	1	1
k1_inh_t	10	10	10	10	1	1	1
k2_inh_t	$0.4e^{-6}$	$7e^{-5}$	$2e^{-6}$	$0.1e^{-4}$	0.1	0.1	0.08
k3_inh_t	1	2	0.02	0.02	0.01	0.01	0.5
rbp_pos	430	455	430	455	480	480	480
rbp_br_t	0.3	0.5	1	2	5	5	5
rbp_e_up	30	30	30	30	1	1	30
rbp_e_down	50	50	50	50	80	80	40
rbp_h_c	8	8	8	8	8	8	8
rbp_inh	1	1	1	1	01	1	1
pol_range	20	20	20	20	20	20	20
k_spl_i	2	2	2	2	2	2	2
k_spl_s	2	2	2	2	2	2	2
k_ret_t	0.001	0.001	0.001	0.001	0.001	0.001	0.001

Supplementary Table 6: Parameters for ODE-resolved and stochastic time-delayed models. Vpol corresponds to polymerase speed, tr_len the length of the transcript. The kX and kX_on parameters provide the initial rate of the reaction and the rate after transcription of the relative feature respectively, with i, s, and esc representing inclusion, skipping, and RBP-mediated inhibition of inclusion respectively.

Parameter	Fig 1	Fig 2 c i	Fig 2 c ii	Fig 2 c iii	Fig 6 a	Fig 6 b	Fig 6 c
vpol	50	50	50	50	50	50	50
tr_len	300	300	300	300	300	300	300
ki	0	0	0	0	0	0	0
ki_on	0.05	0.1	0.1	0.05	0.05	0.1	0.1
ks	0	0	0	0	0	0	0
ks_on	0.5	0.02	0.2	0.005	0.005	0.02	0.2
kesc	0	0	0	0	0	0	0
kesc_on	0	0.5	0.2	0.5	0.5	1	0.2

Supplementary Table 7: Parameter values for the models in figure 7, utilising the model topology specified in supplementary figure 3, and equation 16

Parameter	Figure 7 a, b	Figure 7 c, d	Figure 7 e, f
k_{on}	0.025	0.05	0.01
k_{off}	0.05	0	0.01
v_{syn}	10	10	10
V_{pol}	65	65	65
k_i	0.2	0.1	0.2
k_s	0.1	0.1	0.1
d_i	0.2	0.1	0.2
d_s	0.05	0.1	0.05
tr	10	10	10
Fb_s	0	25	25
K	0	85	45
N	0	9	9
